# Genome wide association analysis of a stemborer egg induced “call-for-help” defence trait in maize

**DOI:** 10.1038/s41598-020-68075-2

**Published:** 2020-07-08

**Authors:** Amanuel Tamiru, Rajneesh Paliwal, Samuel J. Manthi, Damaris A. Odeny, Charles A. O. Midega, Zeyaur R. Khan, John A. Pickett, Toby J. A. Bruce

**Affiliations:** 1 International Centre of Insect Physiology and Ecology (ICIPE), P.O. Box 30772-00100, Nairobi, Kenya; 2 International Crops Research Institute for the Semi-Arid Tropics (ICRISAT), P.O. Box 39063-00623, Nairobi, Kenya; 3 International Institute of Tropical Agriculture (IITA), 5320 Ibadan, Nigeria; 4 School of Chemistry, Cardiff University, Cardiff, CF10 3AT UK; 5 School of Life Sciences, Keele University, Staffordshire, ST5 5BG UK

**Keywords:** Biochemistry, Biotechnology, Chemical biology, Computational biology and bioinformatics, Ecology, Evolution, Genetics, Molecular biology, Plant sciences, Zoology, Ecology, Chemistry

## Abstract

Tritrophic interactions allow plants to recruit natural enemies for protection against herbivory. Here we investigated genetic variability in induced responses to stemborer egg-laying in maize *Zea mays* (L.) (Poaceae). We conducted a genome wide association study (GWAS) of 146 maize genotypes comprising of landraces, inbred lines and commercial hybrids. Plants were phenotyped in bioassays measuring parasitic wasp *Cotesia sesamiae* (Cameron) (Hymenoptera: Braconidae) attraction to volatiles collected from plants exposed to stemborer *Chilo partellus* (Swinhoe) (Lepidoptera: Crambidae) eggs. Genotyping-by-sequencing was used to generate maize germplasm SNP data for GWAS. The egg-induced parasitoid attraction trait was more common in landraces than in improved inbred lines and hybrids. GWAS identified 101 marker-trait associations (MTAs), some of which were adjacent to genes involved in the JA-defence pathway (*opr7, aos1, 2, 3*), terpene biosynthesis (*fps3, tps2, 3, 4, 5, 7, 9, 10*), benzoxazinone synthesis (*bx7, 9*) and known resistance genes (e.g. *maize insect resistance 1, mir1*). Intriguingly, there was also association with a transmembrane protein kinase that may function as a receptor for the egg elicitor and other genes implicated in early plant defence signalling. We report maize genomic regions associated with indirect defence and provide a valuable resource for future studies of tritrophic interactions in maize. The markers identified may facilitate selection of indirect defence by maize breeders.

## Introduction

Plants, being rooted to the ground and unable to flee from attack, have evolved highly sophisticated ways of defending themselves from insect herbivores, over a 400-million-year period of plant-insect interaction and coevolution^1–3^. These defences are wide ranging and can include physical barriers such as lignin, anti-nutritive substances like tannin, production of antibiotics and interactions with associated organisms, which function as natural enemies of the herbivores^2,4^. Some defences are constitutively expressed, whereas others are induced after exposure to herbivore attack, when elicitors from the insect saliva or insect eggs trigger defence responses^5–7^. When plants are exposed to herbivory, they emit herbivore induced plant volatiles (HIPVs)^8–10^. Plants tend to emit larger amounts of volatiles than the insect that is feeding on it and parasitoids and predators have evolved to use HIPVs as cues to locate the herbivores they use as hosts or prey^10–12^. Plant defence involving interaction with the third trophic level is referred to as “indirect defence”, in contrast to direct defences that make the plant less suitable for the herbivore^10^.

While wild plants in natural ecosystems are under natural selection for the ability to defend themselves, domesticated crops have been subjected to artificial selection and are often grown as monocrops^13–15^. These crop plants are vulnerable to attack by adapted insect herbivores, which become pests in this context^16^. Modern maize (corn), *Zea mays* (L.) (Poaceae), was domesticated approximately 9,000 years ago from wild teosinte species^17^. Maize is an essential staple and cash crop for millions of people, particularly in sub-Saharan Africa (SSA). Maize production remains severely constrained by Lepidopteran stemborers, devastating pests of staple cereals in SSA, which reduce yields by up to 80%, depending on the pest population density and the phenological stage of the crop at infestation^18^. Stemborers infest about 50% of the agricultural land in the SSA region, affect the lives of nearly 300 million people and cause yield losses of approximately US$ 1.5 billion per annum^18^.

Nearly all recent commercial maize breeding (artificial selection) has been undertaken in a pesticide treated background^19^. Conventional breeding for host plant resistance against attacking insect pests has largely been done by trial and error or by exposing different genetic lines of crops to the herbivore pests. Potential interactions between different crop genotypes and the natural enemies of the pests have received less attention. We hypothesised that locally adapted varieties (landraces) preferred by smallholder farmers who cannot afford pesticides might have better indirect defence than commercially bred genotypes. Evidence in support of this hypothesis was provided when we discovered that three farmer-selected landraces of maize emitted HIPVs in response to stemborer, *Chilo partellus* (Swinhoe) (Lepidoptera: Crambidae), egg laying whereas the two commercial varieties initially tested did not^7^. The HIPVs emitted by the landraces attracted *Cotesia sesamiae* (Cameron) (Hymenoptera: Braconidae) parasitoid wasps, which are a key natural enemy of the stemborer. These landraces were of South American origin and further studies provided evidence that the egg induced indirect defence trait also exists in some African maize landraces^20^ and in wild teosinte species^21^.

Given these promising initial findings, we embarked on a much larger genome wide association study (GWAS), involving 146 maize genotypes, reported here. The plant trait (phenotype) studied was the ability to “cry for help” by emitting HIPVs to attract *C. sesamiae* parasitic wasp bodyguards after egg deposition by *C. partellus* moths. Our objectives were 1. to determine how widespread this trait was in a wider germplasm collection comprising locally adapted landraces, improved breeding lines and higher yielding commercial varieties, and 2. to develop molecular markers for the indirect plant defence trait. We used GWAS to discover genomic regions and molecular markers associated with it. To the best of our knowledge, this is the first GWAS of parasitoid response to plants induced with insect eggs.

## Results

### Parasitoid attraction to egg-induced volatiles, trait distribution across 146 maize genotypes

A diverse collection of 146 genotypes was tested (Table 1), comprising 9 landraces, 116 inbred lines and 21 hybrids. These were screened, to establish the presence/absence of the egg-induced parasitoid attraction trait. For each genotype, volatiles were sampled, from at least 4 plants with eggs and 4 plants without eggs. Responses of *C. sesamiae* parasitoid wasps to these volatiles were measured in an olfactometer bioassay, with at least 9 parasitoid wasp bioassays per genotype. DNA samples were collected from the same plants (see below). Olfactometer bioassay data are shown in Supplementary Table 1, which details the mean time spent by wasps in the different arms of the olfactometer arm (i.e. arms containing volatiles from plants with eggs; volatiles from plants without eggs, and the mean of the two solvent control arms). Egg-induced parasitoid attraction was observed when wasps spent significantly more time in the “with eggs” zone than in the “without eggs” zone. The trait was normally distributed in the whole population, as well as in the various classification groups (Supplementary Figure 1).

**Table 1:** List of maize genotypes used in the study, ranked in order of attractiveness to *Cotesia sesamiae* parasitoids when induced with *Chilo partellus *eggs.

Rank	Category	Name	Reference code	Rank	Category	Name	Reference Code
1	Landrace	VENEZUELA 648	B4-6E-1	74	Inbred line	CKSPL10021	B13-12E-2
2	Inbred line	CML312	B8-7E-3	75	Inbred line	CML444	B13-9E-2
3	Inbred line	CML442	B8-8E-6	76	Inbred line	CKSPL10112	B14-13E-4
4	Inbred line	CKSPL10089	B14-9E-2	77	Inbred line	EX-HANANAG-4	B18-4E-1
5	Inbred line	CKSPL10090	B14-12E-3	78	Inbred line	WEEVIL WHITE	B17-7E-1
6	Inbred line	M211	B16-8E-1	79	Hybrid	CKIR12009	B2-7E-1
7	Inbred line	MSMP-ZEBRA-2	B17-5E-1	80	Inbred line	SM-706-68	B11-5E-1
8	Inbred line	P300C5S1B	B11-1E-4	81	Inbred line	LPSC7-F64	B9-10E-8
9	Inbred line	P100C6-200	B11-3E-1	82	Inbred line	SAGAM	B17-9E-1
10	Inbred line	CKSPL10036	B14-1E-2	83	Inbred line	CKSBL10043	B13-5E-3
11	Hybrid	MASENO EH-11	B16-2E-1	84	Inbred line	CKSPL10086	B14-11E-1
12	Inbred line	EX614 PX389	B16-4E-1	85	Inbred line	CML445	B9-3E-4
13	Hybrid	CKIR1200-1	B2-1E-4	86	Inbred line	DTPWC9-F104	B9-8E-8
14	Inbred line	X614-3	B16-1E-1	87	Inbred line	CKSPL10191	B15-6E-1
15	Inbred line	CKSPL10042	B14-2E-2	88	Inbred line	EX614PRW	B18-10E-1
16	Inbred line	CKSBL10060	B13-4E-1	89	Inbred line	EX87/312 F4-1	B17-12E-1
17	Landrace	CUBA91	B4-3E-3	90	Inbred line	CZL03007	B9-7E-2
18	Landrace	NYAMULA	B4-5E-6	91	Hybrid	CKIR12011	B3-1E-5
19	Inbred line	CKSBL10027	B8-9E-13	92	Inbred line	MSVTOL-2	B17-6E-4
20	Inbred line	CKSPL10074	B14-3E-3	93	Inbred line	CML443	B9-1E-6
21	Inbred line	X87/02/312 F4-5	B18-1E-1	94	Inbred line	MSMP-ZEBRA	B16-3E-1
22	Inbred line	CKSPL10007	B13-11E-2	95	Inbred line	CKSPL10028	B13-10E-1
23	Inbred line	CKSBL-10038	B12-9E-1	96	Inbred line	ABLEP	B17-3E-1
24	Inbred line	CKSBL10001	B11-2E-8	97	Inbred line	CKSPL10081	B14-5E-4
25	Landrace	HAITI 24	B4-4E-4	98	Inbred line	CKSPL10309	B15-12E-1
26	Landrace	BRAZIL 1006	B8-3E-8	99	Inbred line	CKSPL10341	B15-9E-3
27	Inbred line	CKSBL-10015	B12-1E-2	100	Inbred line	601-STR	B16-6E-1
28	Inbred line	CKSBL-10035	B12-8E-2	101	Inbred line	EX-44/42-2	B17-2E-1
29	Inbred line	CKSBL-10034	B12-7E-1	102	Inbred line	CKSBL-10030	B12-4E-1
30	Inbred line	CKSPL10136	B15-2E-1	103	Inbred line	CML197	B7-4E-8
31	Inbred line	EX614389/F3-1	B18-15E-1	104	Inbred line	X87/02/312-8	B17-10E-1
32	Inbred line	CKSPL10256	B15-10E-1	105	Inbred line	M7P	B18-11E-1
33	Inbred line	ABR	B16-7E-1	106	Inbred line	SM-706-70	B11-8E-2
34	Inbred line	CKSBL10046	B13-3E-1	107	Inbred line	CKSBL-10020	B12-2E-2
35	Inbred line	CKSBL10004	B13-7E-1	108	Inbred line	X87/02/312 F3-2-1	B18-9E-1
36	Inbred line	CKSBL10045	B13-2E-4	109	Hybrid	CKIR12008	B2-6E-4
37	Inbred line	CML334	B7-7E-2	110	Inbred line	CML440	B7-9E-2
38	Inbred line	CKSBL-10033	B12-5E-1	111	Inbred line	CML511	B9-6E-8
39	Inbred line	CKSBL10003	B11-7E-6	112	Hybrid	MASENO EH10	B18-12E-1
40	Hybrid	ET14 MASENO	B18-2E-1	113	Inbred line	EX-218	B17-1E-1
41	Inbred line	CKSPL10146	B15-3E-4	114	Inbred line	CKSPL10186	B15-5E-1
42	Inbred line	CKSBL10007	B11-4E-3	115	Inbred line	EX-YELLOW	B17-8E-1
43	Hybrid	SC-DUMA 43	B13-14E-2	116	Hybrid	CKIR12017	B3-6E-1
44	Inbred line	CKSPL10085	B14-7E-1	117	Inbred line	CZL01005	B9-5E-1
45	Inbred line	CKSPL10273	B15-8E-1	118	Inbred line	F-WHITE	B16-9E-1
46	Inbred line	CKSPL10035	B13-13E-2	119	Landrace	SEFENSI	B4-1E-1
47	Inbred line	SAGAM EX87	B18-7E-1	120	Inbred line	X87/02/312 F4-4	B18-8E-1
48	Inbred line	CKSPL10087	B14-10E-3	121	Inbred line	CKSBL10014	B11-9E-1
49	Inbred line	CKSBL-10040	B12-12E-3	122	Inbred line	CML441	B9-2E-4
50	Inbred line	CKSPL10212	B15-7E-1	123	Inbred line	CKSBL-10021	B12-3E-1
51	Inbred line	XB7/02/312 F4-2DC	B18-14E-1	124	Hybrid	MASENO-EH-12	B16-5E-1
52	Inbred line	CML144	B7-2E-7	125	Landrace	KONGERE	B4-8E-2
53	Hybrid	CKIR12018	B3-7E-1	126	Inbred line	CKSBL-10028	B12-6E-4
54	Hybrid	CKIR12010	B2-8E-2	127	Inbred line	X87/02/312 F4-6	B18-3E-1
55	Inbred line	CKSPL10070	B14-4E-1	128	Inbred line	CML78	B7-1E-8
56	Inbred line	ABR/ABLEP/ABR FS-202	B18-13E-1	129	Inbred line	CML202	B7-5E-7
57	Inbred line	CKSPL10080	B14-6E-4	130	Inbred line	CKSBL10005	B11-6E-1
58	Inbred line	CKSPL10164	B15-1E-2	131	Hybrid	CKIR12004	B2-3E-2
59	Inbred line	CKSBL10013	B13-6E-1	132	Inbred line	CML488	B9-4E-8
60	Inbred line	CKSPL10280	B15-11E-1	133	Inbred line	DTPWC9-F115	B9-9E-3
61	Hybrid	CKIR12013	B3-3E-6	134	Inbred line	EX-6-20R	B16-10E-1
62	Inbred line	CKSBL10042	B13-1E-1	135	Landrace	ENDERE	B4-7E-5
63	Inbred line	CKSBL-10039	B12-11E-3	136	Inbred line	EXT-STR-150	B17-4E-1
64	Inbred line	CKSPL10088	B14-8E-1	137	Landrace	JOWI RED	B4-2E-2
65	Inbred line	EX87/02/312 F3-2-2	B17-11E-1	138	Inbred line	EX-614-PSD	B16-11E-1
66	Hybrid	CKIR12014	B3-4E-4	139	Hybrid	CKIR12016	B3-5E-2
67	Inbred line	CKSPL10170	B15-4E-1	140	Inbred line	X87/02/312F3-3-1	B18-5E-1
68	Inbred line	CML159	B7-3E-8	141	Inbred line	CKSPL10230	B15-13E-1
69	Inbred line	CKSBL-10041	B12-10E-1	142	Inbred line	WEEVIL PURPLE	B18-6E-1
70	Inbred line	CML204	B7-6E-6	143	Hybrid	CKIR12003	B2-2E-1
71	Hybrid	CKIR12012	B3-2E-3	144	Inbred line	CML395	B7-8E-7
72	Hybrid	CKIR12006	B2-4E-1	145	Inbred line	EX 614-F3-2	B16-12E-1
73	Hybrid	CKIR12007	B2-5E-1	146	Hybrid	CKIR12019	B3-8E-1

**Fig. 1:**
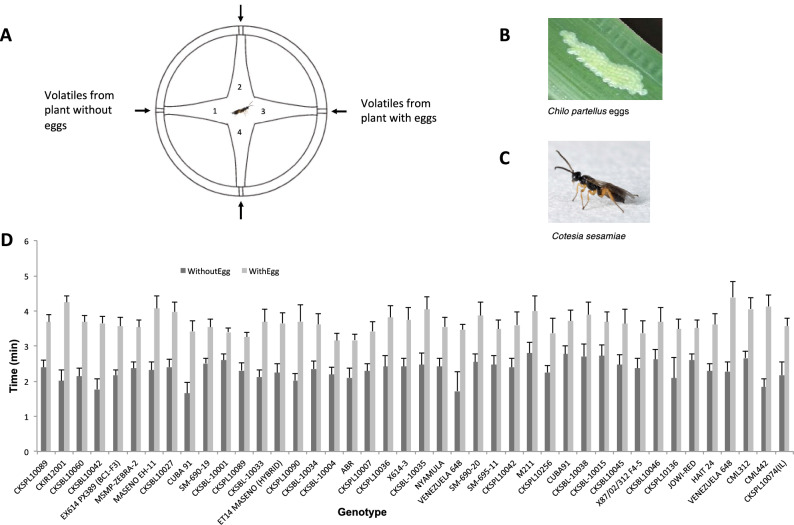
(**A**) Diagram of the 4-arm olfactometer. Insects were allowed to walk freely between 4 discrete odour zones (Zone 1: volatiles from plant without eggs; Zone 3: volatiles from plant with eggs; Zones 2 and 4: solvent blank). Time spent (min) in each zone was recorded. (**B**) *Chilo partellus* eggs. (**C**) *Cotesia sesamiae* parasitoid wasp. (**D**) Olfactometer bioassay response of parasitoid wasp, *C. sesamiae*, to volatiles from maize plants with and without stemborer, *C. partellus*, eggs. Parasitoids could choose between a zone containing volatiles from a plant with eggs (“WithEgg”) and a zone containing volatiles from a plant without eggs (“WithoutEgg”). Mean time spent (min ± SE) is shown for each genotype. Only genotypes that were significantly attractive ($\textit{P} < 0.05$, ANOVA) are shown.

Analysis of variance (ANOVA) revealed significant differences in parasitoid wasp responses ($\textit{P} < 0.05$) for the time spent in the different olfactometer arms for 43 genotypes (Supplementary Table 1). We plotted the means of these 43 genotypes and observed a clear difference between the mean time spent in the olfactometer arm containing volatiles collected from plants with stemborer eggs, in comparison with the controls (Supplementary Figure 2). By comparing mean time spent in “with eggs” and “without eggs” arms, we found 42 genotypes in which the means of observations were significantly different (Table 2) i.e. there was attraction to egg-induced volatiles. Figure 1 shows differences between “with eggs” and “without eggs” observations per plant for these 42 genotypes, of which 6 were landraces, 33 were inbred lines and 3 were hybrids. Landraces, therefore, gave the highest proportion of number of plants having the trait (6 out of 15 screened = 40%) in comparison with inbred lines (33 out of 130 = 25.4%) and hybrids (3 out of 23 = 13%).

**Table 2:** A summary of maize genotypes that were attractive to *Cotesia sesamiae* parasitoids in the olfactometer ($P < 0.05$). Genotype suffixes -1 and -2 indicate different plants of the same genotype. A significant difference in time spent by the parasitoid was observed between “without eggs” and “with eggs” olfactometer arms for these genotypes. Please note full details of all genotypes are provided in Supplementary Table 1.

Genotype	Classification	Population (see Fig. 2)	Mean time (min)		S.E.		P-value
			Without eggs	With eggs	Without eggs	With eggs	
VENEZUELA648-1	Landrace	F	2.28	4.39	0.27	0.44	$<0.0001$
CML312	Inbred line	D	2.66	4.06	0.19	0.31	$<0.0001$
CML442	Inbred line	D	1.84	4.13	0.23	0.32	$<0.0001$
CKSPL10089-1	Inbred line	B	2.39	3.69	0.20	0.21	0.0001
CKSPL10090	Inbred line	B	2.02	3.70	0.20	0.47	0.0001
M211	Inbred line	A	2.80	4.00	0.30	0.42	0.0001
MSMP-ZEBRA-2	Inbred line	A	2.37	3.53	0.19	0.21	0.0001
P300C5S1B	Inbred line	E	2.54	3.87	0.24	0.39	0.0002
P100C6-200	Inbred line	D	2.51	3.53	0.14	0.23	0.0002
CKSPL10036	Inbred line	B	2.42	3.82	0.30	0.33	0.0002
MASENO EH-11	Hybrid	A	2.31	4.07	0.25	0.35	0.0002
EX614PX389 BC1-F3	Inbred line	A	2.17	3.57	0.15	0.26	0.0003
CKIR12001-1	Hybrid	D	2.01	4.25	0.32	0.19	0.0005
X614-3	Inbred line	B	2.42	3.75	0.23	0.35	0.0005
CKSBL10042	Inbred line	B	1.77	3.65	0.29	0.19	0.0006
CKSBL10060	Inbred line	D	2.14	3.69	0.23	0.18	0.0010
CUBA91-1	Landrace	F	1.65	3.42	0.31	0.29	0.0012
JOWI-RED	Landrace	A	2.61	3.52	0.16	0.22	0.0012
NYAMULA-1	Landrace	A	2.45	3.37	0.21	0.21	0.0016
CKSBL10027	Inbred line	C	2.40	3.98	0.23	0.28	0.0017
CKSPL10074	Inbred line	B	2.18	3.57	0.38	0.23	0.0017
X87/02/312 F4-5	Inbred line	A	2.35	3.65	0.25	0.30	0.0019
CKSPL10007	Inbred line	B	2.29	3.42	0.21	0.28	0.0021
NYAMULA-2	Landrace	A	2.42	3.55	0.23	0.28	0.0024
CKSBL10038	Inbred line	C	2.71	3.89	0.36	0.37	0.0024
CKSBL10001	Inbred line	C	2.60	3.40	0.18	0.12	0.0025
CUBA91-2	Landrace	F	2.77	3.71	0.23	0.31	0.0028
HAITI 24	Landrace	F	2.28	3.62	0.22	0.31	0.0045
BRAZIL1006	Landrace	F	2.44	3.54	0.43	0.43	0.0049
CKSBL10015	Inbred line	E	2.72	3.68	0.31	0.28	0.0056
CKSBL10035	Inbred line	B	2.47	4.04	0.34	0.38	0.0070
CKSBL10034	Inbred line	C	2.35	3.62	0.22	0.31	0.0089
VENEZUELA648-2	Landrace	F	1.72	3.47	0.55	0.15	0.0090
CKSPL10136	Inbred line	B	2.09	3.48	0.59	0.28	0.0103
EX614X389 F3-1	Inbred line	A	2.55	3.22	0.21	0.21	0.0119
CKSPL10042	Inbred line	B	2.40	3.60	0.25	0.37	0.0122
CKSPL10256	Inbred line	B	2.25	3.37	0.19	0.43	0.0125
ABR	Inbred line	B	2.10	3.17	0.26	0.16	0.0138
CKSPL10089-2	Inbred line	B	2.30	3.26	0.22	0.13	0.0145
CKSBL10046	Inbred line	B	2.62	3.69	0.28	0.41	0.0150
CKSBL10004	Inbred line	C	2.19	3.15	0.20	0.20	0.0164
CKSBL10045	Inbred line	C	2.47	3.63	0.28	0.43	0.0226
CML334	Inbred line	E	2.26	3.19	0.26	0.26	0.0328
CKSBL10033	Inbred line	C	2.16	3.74	0.33	0.33	0.0384
CKSBL10003	Inbred line	C	2.47	3.49	0.25	0.25	0.0400
ET14 MASENO	Hybrid	A	2.36	3.37	0.29	0.34	0.0480

### SNP discovery, distribution, heterozygosity and linkage disequilibrium

Genotyping-by-sequencing (GBS) data were generated from 1018 individual maize plants (4-6 replicates per genotype), which were representative of 146 diverse accessions (Table 1). In total, 2.1 billion reads were generated at an average of 2.06 million reads per maize genotype. We called 316,127 (0.32M) raw SNPs from all the plants genotyped, and later filtered the raw SNPs to 54,311 (54K) for subsequent analysis. The distribution of the 54K SNPs across the maize genome is shown in Supplementary Figure 3 and Supplementary Table 2. The number of SNPs per chromosome ranged from 3748 (Chr10) to 9275 (Chr1). The filtered SNPs resulted in an average marker density of 27 SNPs/Mbp of the maize genome. The average heterozygosity proportion for the whole maize population was 0.048 but was higher in hybrid (0.094) and landrace (0.080) subpopulations and lower in the inbred subpopulation (0.037). Linkage disequilibrium (LD) and LD decay distance in the 10 maize chromosomes are summarised in Supplementary Figures 4 and 5. The average whole genome LD decay is shown in Supplementary Figure 4. The genetic distance at which the estimated $\hbox {R}^{2}$ fell below 0.2 ranged from 0.9 kb to 1 kb in all the 10 maize chromosomes except chromosomes 4 and 8 (Supplementary Figure 5). LD decay for chromosomes 4 and 8 ranged from 1 kb to 1.5 kb at $\hbox {R}^{2} < 0.4$.

**Fig. 2:**
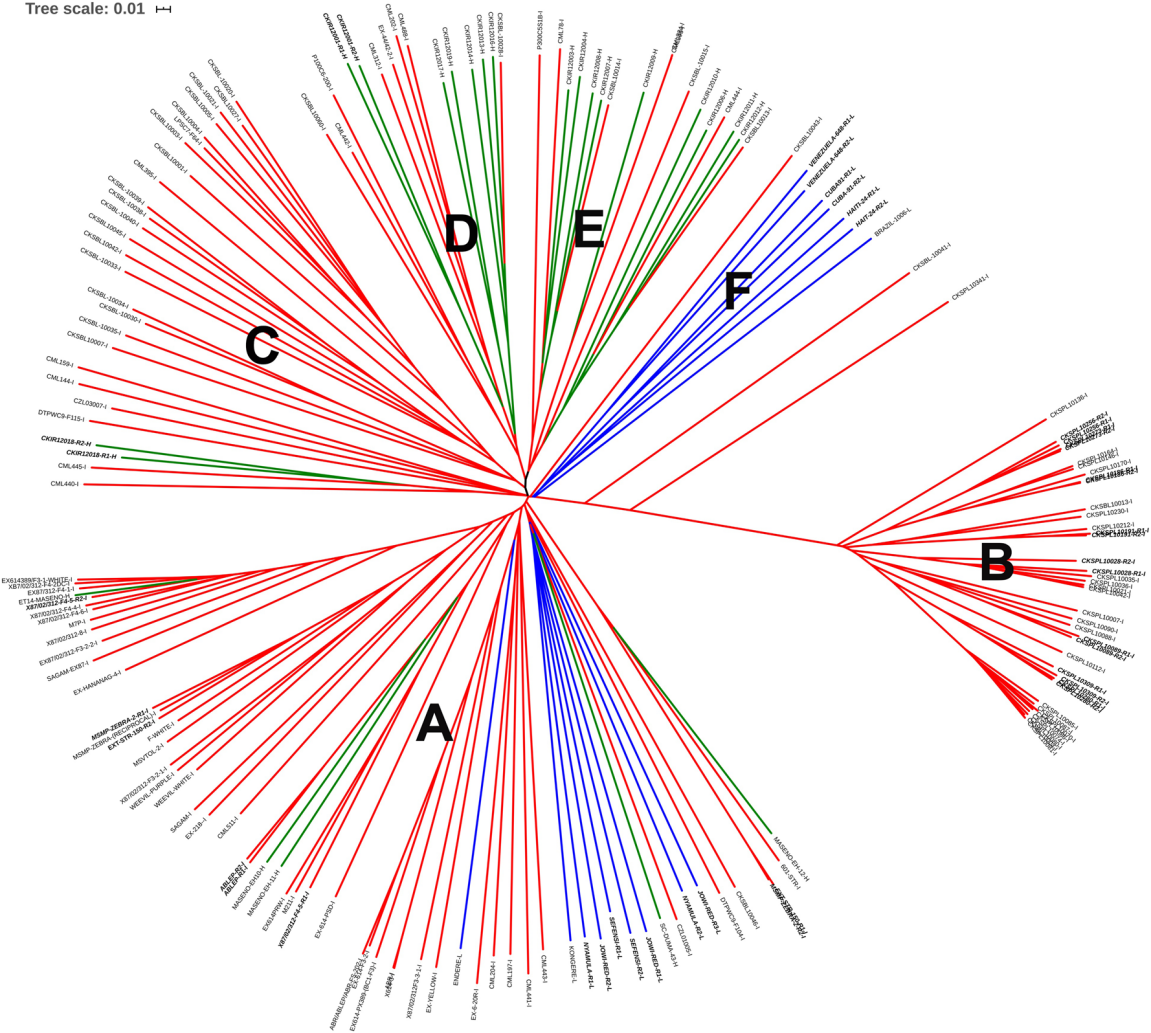
Phylogenetic tree showing genetic diversity of the maize genotypes using neighbor-joining method. Scale represents genetic distance: 0.1 is 10% genetic difference between genotypes. Blue, green and red lines represent landraces, hybrids and inbred lines respectively (also denoted by -L, -H and -I suffixes on genotype names). Genotypes in bold indicate where genetic separation was found within a genotype. Clusters A–F represent discrete genetic groups with similar pedigree and origin.

### Genetic diversity and population structure in the maize population

A similarity cladogram across the maize genotypes revealed 6 clusters (Fig. 2), generally grouped according to their pedigree and origin. Cluster A was composed mainly of landraces, inbred lines and hybrids developed at a local breeding program in Kenya (Maseno University). There was distinct clustering of inbred lines whose names started with the acronym CKSPL (Fig. 2, Cluster B). Landraces from Cuba, Brazil, Haiti and Venezuela clustered together (Cluster F). The rest of the genotypes clustered according to their pedigree and breeding history. We observed significant genotypic differences among plants of two landraces (“Nyamula” and “Jowi-red”) and four inbred lines (Ext-STR-150, MSMP-ZEBRA-2 and X87/02/312-F4-5, CML-395). Each genetically diverse plant was further treated as an independent genotype, bringing the total number of distinct genotypes used for genetic analysis to 167 lines. Furthermore, principal component analysis (PCA) confirmed a similar pattern of genetic diversity among the maize genotypes (Supplementary Figure 6) with the first three principal components (PC1, PC2 and PC3) explaining 14.5%, 5% and 3.2% of total genetic variance respectively. The ADMIXTURE model (K = 6) also predicted an optimum population number of 6 (Supplementary Figure 7). An admixed population is one that has multiple ancestral genetic proportions i.e. there is evidence of outbreeding. Populations A and D consisted mainly of inbred lines and advanced crosses being used in the Maseno University breeding program. Other than populations A, B and D, in which the accessions did not have significant admixtures, the rest of the clusters comprised mainly of admixed populations (Supplementary Figure 7). The genotypes that were considered to have the indirect defence trait did not cluster in any preferential manner but were distributed across the various populations.

### Marker-trait associations

**Fig. 3:**
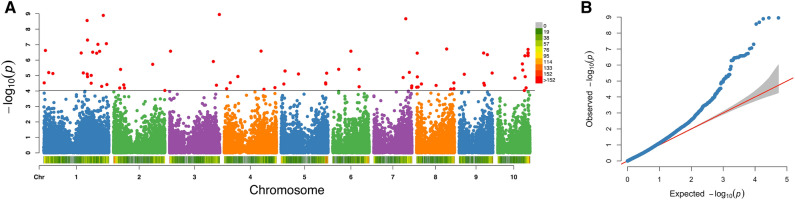
(**A**) Manhattan plot using MLM approach indicating SNPs significantly associated with the egg induced parasitoid attraction trait (shown in red). SNP density is indicated by the colour scale on the bar next to the X-axis (scale given in inset). The X-axis is the genomic position of the SNPs in the genome, and the Y- axis is -log_10_ of the P-values. Each chromosome is coloured differently. The grey horizontal line represents the minimal significant level at the cutoff of FDR 0.05 (*MLM* Mixed Linear Model, *SNP* Single Nucleotide Polymorphism, *FDR* False Discovery Rate) (**B**) Quantile-quantile plot.

A total of 101 significant SNP-trait associations were identified (Supplementary Table 3) using both GLM + PCA and MLM + PCA + K analysis approaches, after FDR correction (q-value of 0.05)(GLM = General Linear Model, PCA = Principal Component Analysis, MLM = Mixed Linear Model, K analysis = Cluster analysis, FDR = False Discovery Rate). The *P*-value threshold was 9.23 × 10^-5^. The Manhattan plot of associated SNPs (for parasitoid wasp response to stemboer egg-induced plant volatiles, analysed with the 54,311 SNPs) is shown in Fig. 3 for MLM + PCA + K analysis, and Supplementary Figure 8 for GLM + PCA analysis. All 101 identified SNPs were located across all 10 maize chromosomes. More than half of the significant markers were located on chromosomes 1 (21 SNPs), 5 (12 SNPs), 8 (10 SNPs) and 10 (15 SNPs) (Supplementary Table 3). The QQ plots (Fig. 3 and Supplementary Figure 8) revealed that both GL and ML models successfully controlled any false positive associations that may have resulted from underlying population structure. The phenotypic variation ($\hbox {R}^{2}$) explained by the associated SNPs in GLM and MLM approach ranged from 0.099 – 0.498 and 0.123 – 0.409, respectively. These high $\hbox {R}^{2}$ values and their consistency in both GLM and MLM approaches provide more confidence to the identified SNPs and are an indication that the association is not merely by chance.

**Table 3:** List of 33 candidate maize genes located within 10 mb of 23 SNPs significantly associated with the indirect defence trait. *Chr* chromosome. More details about these candidate genes, including their exact locations and web link addresses, are provided in Supplementary Table 4

No.	Chr	SNP position	GLM + PCA			MLM + PCA + K			Candidate gene	Distance from SNP (mb)	Gene model name in maize database
			*P*-value	FDR adjusted *P*-value	Marker-R$^{2}$	*P*-value	FDR adjusted *P*-value	Marker-R$^{2}$			
1	1	182904826	2.69E-07	7.34E-03	0.1766	6.74E-06	0.02105	0.1521	*bx9*	2.5	Zm00001d031209
2	1	200500239	3.56E-18	7.34E-03	0.4985	2.73E-09	0.02105	0.4096	*opr7*	7.4	Zm00001d032049
									*hm1*	0.75	Zm00001d031802
3	1	219232870	1.72E-07	7.34E-03	0.2065	3.12E-05	0.02813	0.1541	*tps7*	4.3	Zm00001d032230
4	1	23589496	1.08E-09	7.34E-03	0.3034	6.38E-06	0.02105	0.2278	*aos2*	5.3	Zm00001d028282
5	1	275027100	6.38E-14	7.34E-03	0.4060	1.28E-09	0.02105	0.3657	*aos3*	5.82	Zm00001d034186
6	1	289271440	2.63E-12	7.34E-03	0.3542	8.63E-08	0.02105	0.2798	*bm2*	2.81	Zm00001d034602
7	3	202720889	1.68E-09	7.34E-03	0.2743	1.23E-06	0.02105	0.2241	*fps3*	2.4	Zm00001d043727
8	3	216989090	7.39E-08	7.34E-03	0.2071	4.21E-05	0.03043	0.1533	*srph1*	0.4	Zm00001d044172
9	3	230441690	7.39E-08	7.34E-03	0.20718	4.21E-05	0.03043	0.1532	*pme31*	1.72	Zm00001d044585
10	4	11266704	2.47E-06	7.34E-03	0.1518	7.09E-05	0.02105	0.1358	*cystatin9*	4.05	Zm00001d049111
11	4	28116948	5.29E-08	7.34E-03	0.2367	2.89E-05	0.02798	0.1763	*bx7*	9.8	Zm00001d049179
12	5	80439617	6.52E-08	7.34E-03	0.2255	7.92E-06	0.02105	0.1506	*tps2*	9.0	Zm00001d015053
									*tps3*	9.0	Zm00001d015054
13	6	84376158	5.65E-14	7.34E-03	0.3608	2.65E-07	0.02105	0.2701	*mir1*	4.5	Zm00001d036542
									*mir2*	4.5	Zm00001d036541
									*chn2*	1.51	Zm00001d036370
									*cdpk13*	0.0007	Zm00001d036416
									*mpk15*	1.74	Zm00001d036448
14	6	122394808	1.53E-07	7.34E-03	0.2436	5.28E-05	0.03422	0.1496	*px5*	2.7	Zm00001d037550
15	7	147575520	9.01E-15	7.34E-03	0.4192	2.11E-09	0.02105	0.3576	*ccp4*	5.1	Zm00001d021615
16	8	3163970	6.63E-12	7.34E-03	0.36848	3.54E-07	0.02105	0.2796	*cystatin4*	1.8	Zm00001d008268
17	8	138888278	2.11E-12	7.34E-03	0.34187	1.91E-07	0.02105	0.26444	*mmt1*	3.83	Zm00001d011099
18	8	157671586	4.16E-04	0.040	0.1271	7.48E-05	0.04035	0.1501	*cystatin10*	4.9	Zm00001d012068
19	8	172187113	4.08E-06	7.34E-03	0.17647	7.07E-05	0.04103	0.1523	*cystatin2*	0.8	Zm00001d012561
20	9	152534623	1.09E-07	7.34E-03	0.1937	6.86E-06	0.02105	0.1753	*aos1*	7.1	Zm00001d048021
									*LRR-RLK*	0.27	Zm00001d048390
21	10	80491988	1.78E-07	7.34E-03	0.1888	1.46E-05	0.02164	0.1675	*tps10*	6.0	Zm00001d024486
									*tps4*	6.4	Zm00001d024478
									*tps5*	6.2	Zm00001d024481
									*tps9*	6.6	Zm00001d024477
22	10	116675865	1.11E-09	7.34E-03	0.2454	4.42E-06	0.02105	0.2349	*scp1*	3.6	Zm00001d025526
23	10	126494378	5.39E-11	7.34E-03	0.32567	5.27E-07	0.02105	0.33411	*pme1*	4.26	Zm00001d025588

We retrieved 33 candidate genes (Table 3) within 10 Mbp up- and downstream of 23 associated SNP QTL (quantitative trait locus) positions using the ZmB73 RefGen v2 database (https://www.maizegdb.org/gbrowse). These genes have previously been annotated with a plant defence function. The phenotypic variation ($\hbox {R}^{2}$) of the 23 associated SNPs ranged from 0.099 – 0.498 with the GLM + PCA approach and 0.123 – 0.409 with the MLM + PCA + K approach. The distance between the 33 candidate genes and SNP positions ranged from 0.0007 mb (*cdpk13* gene) to 9.8 mb (*bx7* gene). Detailed information about candidate genes and their roles in plant defence is given in Supplementary Table 4. We also provide, in Supplementary Table 5, a listing of 202 genes located within a 10 mb region of the top 16 SNPs (selected based on having an $\hbox {R}^{2}$ value of above 25% (0.25) with the MLM approach).

## Discussion

Multitrophic interactions with natural enemies of herbivores allow plants to increase herbivore mortality by recruitment of “bodyguards” after changing their volatile emission profile to become more attractive to the natural enemies^12,22^. This “call for help” signalling is known as indirect defence^10^. The genetic basis for variation in insect egg-induced indirect defence between crop genotypes is poorly understood and therefore the current study was designed to identify regions of the maize genome associated with it, using *C. partellus* as the herbivore and *C. sesamiae* as the natural enemy. Our current study bridges the gap between studies of the chemical ecology of multitrophic interactions and plant genomics. Our previous studies^7,20^ showed that certain maize landraces responded to egg laying, the earliest stage of attack by maize stemborer, *C. partellus* insects, by emitting volatiles attractive to parasitoid wasps that are key natural enemies of the herbivore. However, this indirect defence trait was absent in the limited number of improved hybrids we initially tested. Here we provide a much larger analysis of 146 maize genotypes, comprising landraces, inbred lines and commercial hybrids, in a genome wide association study (GWAS).

Our earlier studies^7,20^revealed the suite of plant volatiles induced by *C. partellus* eggs in maize. Thus, identification of the HIPVs was not the focus of the current study. Volatile samples in the current study were analysed by gas chromatography (data not shown) and similar key compounds, in particular (3*E*)-4,8-dimethyl-1,3,7-nonatriene (DMNT), were induced. Here we aimed to identify molecular markers for parasitoid attraction and obtain insight into adjacent potential candidate genes underpinning this indirect defence trait. Availability of molecular markers, provided in the current study, could facilitate accelerated breeding for improved maize cultivars with the indirect defence trait through marker assisted selection (MAS). We used the parasitoid bioassay response itself to directly measure parasitoid attraction, rather than use a proxy in terms of HIPVs. Our study used a biodiverse collection of maize genotypes, which were exposed to *C. partellus* eggs prior to volatile collection. Volatile samples were then used in large scale parasitoid bioassays, for all 146 genotypes, in a choice test, testing if volatiles from egg exposed plants were significantly preferred to volatiles from unexposed control plants. We found the indirect defence trait was more widespread in landrace germplasm (40% of genotypes) but, because these were not genetically uniform, considerable variation between individual plants was observed. The trait was found in 25% of inbred lines and in 13% of hybrids. These lines were more consistent because they were genetically uniform. Furthermore, discovering the indirect defence trait in improved lines opens up the prospect of introgressing the trait into other higher yielding maize cultivars with desirable agronomic characteristics.

Data were subjected to a GWAS analysis which revealed 101 SNPs strongly associated with the trait. Within a 10mb region of the genome next to these SNPs, there were 33 candidate genes that may code for the trait. Of these, 7 are terpene synthase genes (*tps2*, *tps3*, *tps4*, *tps5*, *tps7*, *tps9* and *tps10*). This is not surprising because the indirect defence trait operates by emission of volatiles. Previous studies have linked terpene synthases to indirect defence^23,24^. Genes implicated in DMNT emission, induced by a synthetic jasmonic acid (JA) analogue, were investigated in an earlier GWAS by Richter *et al.*^24^ who found a strong association with *tps2*. *Farnesyl diphosphate synthase3* (*fps3*) is another candidate gene and catalyses biosynthesis of precursor molecules for terpene biosynthesis. Several of our other candidate genes are implicated in plant secondary metabolism. The most notable of these are *12-oxo-phytodienoic acid reductase7* (*opr7*), *allene oxide synthesis1* (*aos1*) *allene oxide synthesis2* (*aos2*) and *allene oxide synthesis3* (*aos3*) which encode key enzymes in the JA-defence pathway^25,26^. Another candidate gene that potentially plays a role is *methionine S-methyltransferase1* (*mmt1*) as methyltransferases can be involved in plant volatile biosysnthesis^27^.

To trigger the plant defence cascade culminating in release of herbivore induced volatiles, the plant needs to detect the presence of the insect eggs through molecular recognition of the egg elicitor. A putative receptor gene, GRMZM2G438840, is strongly associated with the trait. It is annotated as a leucine-rich repeat transmembrane protein kinase family protein and was identified by^28^ as a putative immune receptor gene. A topic for future studies would be to investigate if silencing this gene prevents molecular recognition of *C. partellus* eggs. A clade I L-type lectin receptor kinase LecRK-I.8 has recently been shown to be involved in detection of *Pieris brassicae* insect eggs in Arabidopsis^29^. There is also a *chitinase2* (*chn2*) which could play a role in interactions with eggs that contain chitin.

Two of our candidate genes are implicated in early plant defence signalling: *calcium dependent protein kinase13* (*cdpk13*) has been shown to be a component of touch- and wound-induced pathways involved in early stages of local and systemic responses in maize^30^. Calcium-dependent protein kinases (CDPKs) play a vital role in stress signalling by detecting increases in $\hbox {Ca}^{2+}$ and transducing them into phosphorylation events^31^. We also found a mitogen-activated protein kinase, *MAP kinase15* (*mpk15*), associated with the indirect defence trait. Reducing the function of MAP kinases has been reported to impair the synthesis of secondary stress signals, including JA, and loss of MAPK function results in reduced resistance of plants to herbivorous insects^32^. It thus seems plausible that *cdpk13* and/or *mpk15* play a role in egg-induced signal transduction. We also found two pectin methylesterases (PMEs) - *pectin methylesterase1* (*pme1*) and *pectin methylesterase31* (*pme31*). These are noteworthy because PMEs are involved in cell wall modification and pectin catabolic processes^33^.

Interestingly, some of our candidate genes are associated with direct defence. These include *maize insect resistance1* (*mir1*) and *maize insect resistance2* (*mir2*) which encode cysteine proteinases (key defensive proteins against chewing insect pests in maize)^34^; *benzoxazinone synthesis7* (*bx7*) and *benzoxazinone synthesis9* (*bx9*), genes for benzoxazinoid biosynthesis^35^, and *brown midrib 2* (*bm2*) which is associated with lignin synthesis^36^, a physical defence. Although we used an unbiased approach in selecting SNPs via the GWAS procedure, the selection of candidate genes was limited by searching for genes annotated with defence functions and it is likely that more genes are known for direct defence than are currently known for indirect defence. Another explanation is that plants that can recognise insect eggs have a suite of defences that are triggered upon detection of eggs which include direct as well as indirect defences. It is possible that genes encoding direct and indirect defences could cluster together in the genome but this would require further study.

Given the above candidate genes, we would like to suggest a hypothetical model by which the egg sensitive maize genotypes respond: Firstly, there is a molecular recognition process by which the *C. partellus* egg elicitor is detected; secondly, the JA-defence pathway is triggered, and, thirdly, JA-associated defences, including HIPV emission are triggered. Thus, the egg sensitive genotypes elicit a suite of defences following stemborer oviposition that comprise both direct and indirect defences which will protect the plant against caterpillars emerging from the eggs.

The SNP molecular markers we have identified provide a resource for future studies of the underpinning genetics involved in indirect defence. We have highlighted regions of the genome associated with parasitoid attraction and have identified candidate genes already annotated with plant defence functions. However, it is likely that there are further genes, not yet annotated, that play a role. A particularly interesting opportunity is to discover a plant receptor used for recognition of the egg elicitor. Novel genes could be discovered that play a role in plant signal recognition, particularly of small lipophilic molecules reviewed in^37^, and biosynthetically related to the egg elicitor (currently under structural elucidation by some of the authors here). Thus, we hope our dataset will allow identification of novel genes involved in indirect defence signalling between maize plants provoked by herbivore (*C. partellus*) eggs and parasitoid wasp “bodyguards” that have not previously been annotated as having roles in plant defence. We provide information (in Supplementary Table 5) about genes in areas of the maize genome in the vicinity of the top 16 SNPs most closely associated with the indirect defence trait.

There are global pressures to reduce pesticide use in agriculture and in any case few African smallholder farmers in the study region have access to pesticides. The current findings will help develop improved maize varieties with indirect defence against stemborers because we have already identified improved lines and hybrids possessing the trait. Preliminary field trials indicate an increase in parasitism of maize stemborers in genotypes with the indirect defence trait. The indirect defence trait was rarer in improved lines than in landraces, perhaps because selection for yield and quality in commercial crop breeding environments could have compromised defence traits because the value of any defence traits would not be realised when plants were treated with insecticide^38,39^. However, it was less rare than expected. Our current findings open up the prospect of breeding crops that enhance biological control of insect pests by natural enemies, such as *C. sesamiae*, through marker assisted selection (MAS). For example, the CIMMYT ESA hybrid maize breeding program is mainly based on four parental lines (CML444, CML395, CML312 and CML442)^40^. We found that CML312 and CML442 possess the egg-induced parasitoid attraction trait, whereas CML395 and CML444 do not. Therefore our study identifies germplasm that could be used to introgress the trait into improved crops. Such crops would be more resilient to insect attack, difficult for insect to develop resistance and less dependent on pesticide application. They would, however, require natural enemies of pests to be present in the agricultural ecosystem as an ecosystem service. A recent meta-analysis^41^ found that “top-down” control of herbivorous insect populations by natural enemies is at least as important as “bottom-up” control by the plant and, thus, breeding crops for increased tritophic interaction with natural enemies^42^ could be a promising approach. Future work should investigate if the genetics identified in the current study with *C. partellus* stemborers is also involved in indirect defence against a new threat to maize in Africa—the invasive fall armyworm, *Spodoptera frugiperda*.

## Methods

### Plant material

A diverse collection of 146 maize genotypes comprising 9 landraces, 116 inbred lines and 21 commercial hybrid varieties were obtained from local farmers (farmer preferred landraces), Maseno University (Kenya), the International Maize and Wheat Improvement Center (CIMMYT, Nairobi, Kenya) and commercial seed suppliers (Table 1). Plants were grown individually in pots filled with fertilised soil in an insect-proof screen house at *icipe*-Thomas Odhiambo campus (ITOC), Mbita Point (0°25’S, 34°12’E; c. 1200 m above sea level), western Kenya. All plants were grown under natural conditions (c. 25 °C, 65% RH, 12L:12D).

### Insects

Field-collected *C. partellus* were reared on a semi-synthetic diet containing sorghum (*Sorghum bicolor*) leaf powder^43^. The larval parasitoid *C. sesamiae* was reared on stemborer larvae using methodologies described previously^44^. Experimental insects were maintained at the insect mass rearing unit of *icipe*-Thomas Odhiambo campus ($24 \pm 3$ °C, $70 \pm 5$% RH, 12L: 12D). The insect culture was infused with field-collected insect population every 3 months to avoid genetic decay and maintain the original behavioural characteristics of the species. Naïve, 1-day old mated female parasitoids obtained from the fourth to fifth generation were used in experiments.

### Volatile collection

Volatile compounds from whole maize plants, with and without stemborer eggs, were collected by headspace sampling^7^. Volatiles were collected from at least 4 plants with and 4 plants without eggs per genotype. Prior to volatile collection, 4-week old maize seedlings were placed inside oviposition cages ($80 \times 40 \times 40$ cm) into which six gravid female stemborer moths were introduced and kept overnight for oviposition. Concurrently, control plants were kept inside similar cages, but without stemborer moths. Volatiles were collected the following day, starting from the last 2 h of photophase, for 24 h. Leaves of plants with or without eggs were enclosed in polyethyleneterephthalate (PET) bags (volume 3.2 L, $\simeq$ 12.5 mm thickness) heated to 150 °C before use and fitted with Swagelock inlet and outlet ports. Charcoal-filtered air was pumped (500 mL min^−1^) through the inlet port. Volatiles were collected on Porapak Q (0.05 g, 60/80 mesh; Supelco, Bellefonte, PA, USA) filters inserted in the outlet port through which air was drawn at 300 mL min^−1^. After entrainment, volatiles were eluted with 0.5 mL dichloromethane (Sigma Aldrich) for use in subsequent bioassays. Volatiles were collected from 1,168 plants representing 146 genotypes.

### Olfactometer bioassay

To phenotype the egg-induced indirect defence trait, behavioural responses of parasitoids to volatiles from different maize genotypes were tested in a Perspex four-arm olfactometer (Fig. 1) described in^7^. Air was drawn through the four arms towards the centre at 260 mL min^−1^. Headspace samples (10 μL aliquots) were applied, using a micropipette (Drummond “microcap”, Drummond Scientific Co., Broomall, PA, USA), to a piece of filter paper (4 × 25 mm) subsequently placed in an inlet port at the end of each Olfactometer arm. Mated female parasitoids, without previous exposure to plants or hosts, were transferred individually into the central chamber of the Olfactometer using a custom-made piece of glass tubing. Time spent in each olfactometer arm was recorded with “Olfa” software (F. Nazzi, Udine, Italy) for 12 min.

The experiments were replicated 9 - 15 times per plant. A choice test was carried out to compare insect responses to headspace samples from oviposition-induced and control plants for all 146 maize genotypes. The two opposite arms held the test stimuli (10 μL aliquots of headspace sample) that had been collected from plants that had stemborer eggs and those without the eggs (see Fig. 1). This dose was approximately equal to the amount emitted by 12 plants over 10 min^7^. The remaining two arms were solvent controls. For each plant, we calculated the average proportion of time spent by the parasitoid in each olfactometer arm across all replications and compared the means using analysis of variance (ANOVA). The means from the two arms representing the solvent controls were analysed together. Comparisons were made: 1. between time spent in arms containing volatiles from solvent control and from a plant with eggs, and 2. between time spent in arms containing volatiles from “with eggs” and “without eggs” plants. Significant observations were determined using *P* $\le$ 0.05. Means of significant observations were separated using Fisher’s LSD test with $\alpha$ set at 0.05 (Genstat version 10, VSN International, Hemel Hempstead, UK). An attraction index was calculated by dividing proportion of time spent in the treated olfactometer area by time spent in the solvent blank control area and log_10_ transforming the data. These attraction index values were used to draw normal distribution curves using the ggplot2 package in R studio (Version 1.1.383) (Supplementary Figure 1). The calculated attraction index value was used for GWAS.

### DNA extraction and genotyping

Fresh leaf samples were collected from assayed plants, immersed in liquid nitrogen, and crushed into fine powder using mortar and pestle. DNA was extracted from 1018 plants (146 maize genotypes, 4-6 plants per genotype) (Table 1) using the DNeasy mini kit (Qiagen, Hilden, Germany), according to manufacturer’s instructions, from at least four individuals per genotype. Purity and quantity of the extracted DNA was determined using gel electrophoresis and a Qubit 2.0 Fluorometer (Life Technologies, Carlsbad, CA, USA) respectively with final dilution to 30 ng/μL. The DNA was sent to Cornell University for library construction (ApeKI restriction enzyme) and genotyping-by-sequencing (GBS). The resulting raw reads were processed using the GBS pipeline of the Trait Analysis by aSSociation, Evolution and Linkage (TASSEL) 5.0 program^45^. Raw SNPs were further filtered using a minor allele frequency of $\ge$ 0.05, minimum depth coverage of 5, maximum mismatch of 3 for alignment, and maximum missing data of 30%. Chromosomal assignment and position of SNPs on the physical map was deduced from the draft whole B73 genome sequence of ZmB73 RefGen v2^46^. SNPs were designated based on chromosome number and position (e.g. Chr1_187669221 meaning SNP located at 187669221th position on chromosome 1).

### Genetic fidelity, diversity, population structure and Genome Wide Association Study

A filtered SNP dataset was used for all molecular analysis in this study. Genetic fidelity was confirmed with identity-by-state distance matrix in Tassel 5.0. We used the filtered SNP data set to generate a Neighbor-Joining cladogram and estimated principal component analysis (PCA) with covariance and five components. The population structure of the genotyped plants was determined using the admixture model with correlated allele frequencies. The estimated proportions of each individual’s genome originating from each of the K ancestral populations (q) was calculated for K ranging from 1 to 10 ancestral populations (or clusters), with 10 runs for each K value. The structure harvester program was used to estimate optimum K value from admixture analysis results^47^. Linkage disequilibrium ($\hbox {R}^2$) was calculated from TASSEL 5.0 and LD decay plot generated using the R-program (http://www.R-project.org/)(version 3.6.2). Association mapping based on General Linear Model (GLM) with PCA as the fixed effect (GLM+PCA); and Mixed Linear Model (MLM) with PCA results and Kinship value (MLM+PCA+K) were conducted in TASSEL 5.0 software. The p values for each marker were adjusted for false discovery rate (FDR) or transformed to q-values using the R package (q-value)^48^. The q-value package has been widely adopted to control for multiple testing^49,50^. We used the positions of significant markers that had a positive effect on the trait as reference points and identified candidate genes falling within 10 Kbp up- and downstream from them on the database (https://www.maizegdb.org/gene_center/gene) of the maize reference genome, ZmB73 RefGen v2 (https://www.maizegdb.org/gbrowse). The selection of these candidate genes was limited by searching for genes annotated with defence functions. In addition, we selected, regardless of any existing annotation, the top 16 SNPs that had an $\hbox {R}^2$ value of $\ge$ 25% (using the MLM approach) out of 101 trait associated SNPs. A total of 202 candidate genes were identified within a 10 mb region of these top 16 SNPs that are closely linked with the indirect defense trait across 10 chromosomes of the maize genome (B73 RefGen v2 maize database).

## Supplementary information

Supplementary file1 (DOCX 2626 kb)

Supplementary file2 (DOCX 137 kb)
