# Supplementary material for: Genome wide association analysis of a stemborer egg induced “call-for-help” defence trait in maize
Source: Sci Rep. 2020 Jul 8;10:11205. doi: 10.1038/s41598-020-68075-2 (PMC7343780; doi:10.1038/s41598-020-68075-2)
Supplement: Supplementary file 1 — Supplementary file1 (DOCX 2626 kb) [file 41598_2020_68075_MOESM1_ESM.docx]

**Genome wide association analysis of a stemborer egg induced “call-for-help” defence trait in maize**

**Amanuel Tamiru**1,+**, Rajneesh Paliwal**2,3,+**, Samuel J. Manthi**2**, Damaris A. Odeny**2**,**  **Charles A.O. Midega**1**, Zeyaur R. Khan**1**, John A. Pickett**4**, and Toby J.A. Bruce**5,*

1International Centre of Insect Physiology and Ecology (ICIPE), P.O. Box 30772-00100, Nairobi, Kenya

2International Crops Research Institute for the Semi-Arid Tropics (ICRISAT) P.O. Box 39063–00623, Nairobi, Kenya

3International Institute of Tropical Agriculture (IITA), PMB-5320, Ibadan, Nigeria

4School of Chemistry, Cardiff University, Cardiff, CF10 3AT, UK

5School of Life Sciences, Keele University, Staffordshire, ST5 5BG, UK

*t.j.a.bruce@keele.ac.uk

+these authors contributed equally to this work

**Supplementary Figure 1.** Distribution of the indirect defence trait in the whole population (**a**), among landraces only (**b**), inbred lines only (**c)** and hybrids (**d**) of maize *Zea mays* (L.) (Poaceae). The trait studied was the ability to emit HIPVs (Herbivore Induced Plant Volatiles) to attract *C. sesamiae* parasitic wasp bodyguards after egg deposition by *C. partellus* moths.

**Supplementary Figure 2.** Olfactometer bioassay response of *Cotesia sesamiae*, a key parasitoid natural enemy of stemborers, to *Zea mays* (L.) volatiles in the olfactometer (Time spent, minutes) for genotypes that were significantly attractive (L = Landrace, IL = Inbred line, H = Hybrid). Parasitoids were presented with zones containing volatiles from a plant with eggs, a zone containing volatiles from a plant without eggs and solvent control.

**Supplementary Figure 3.** Distribution of the 54K SNPs across the maize genome. Scale indicates SNP density.


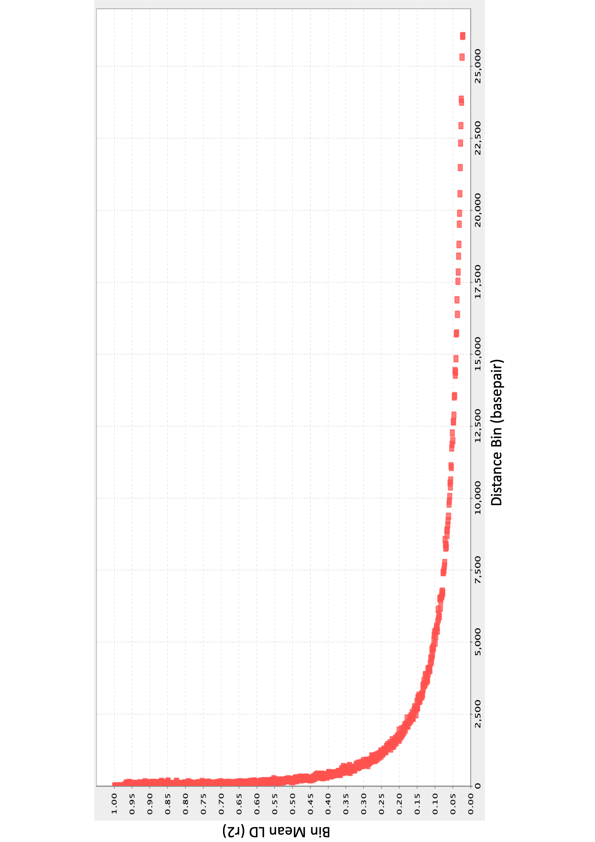


**Supplementary Figure 4.** Average whole genome linkage disequilibrium (LD) decay.

**Supplementary Figure 5.** Linkage disequilibrium (LD) decay distance for the 10 maize chromosomes

**Supplementary Figure 6.** Principal component analysis of maize population; (**a**) PC1 vs PC2, and (**b**) PC1 vs PC3.

**Supplementary Figure 7.** Populations (A-F) predicted from ADMIXTURE model. These are also shown in the cladogram (Fig. 2).

**Supplementary Figure 8.** (**a**) Manhattan plot using GLM approach indicating SNPs significantly associated with the egg induced parasitoid attraction trait (shown in red). SNP density is indicated by the colour scale on the bar next to the X-axis (scale given in inset). The X-axis is the genomic position of the SNPs in the genome, and the Y- axis is -log10 of the P-values. Each chromosome is coloured differently. The grey horizontal line represents the minimal significant level at the cutoff of FDR 0.05. (GLM = General Linear Model, SNP = Single Nucleotide Polymorphism, FDR = False Discovery Rate) (**b**) Quantile-quantile plot
