# Supplementary material for: Genome wide association analysis of a stemborer egg induced “call-for-help” defence trait in maize
Source: Sci Rep. 2020 Jul 8;10:11205. doi: 10.1038/s41598-020-68075-2 (PMC7343780; doi:10.1038/s41598-020-68075-2)
Supplement: Supplementary file 2 — Supplementary file2 (DOCX 137 kb) [file 41598_2020_68075_MOESM2_ESM.docx]

**Genome wide association analysis of a stemborer egg induced “call-for-help” defence trait in maize**

**Amanuel Tamiru**1,+**, Rajneesh Paliwal**2,3,+**, Samuel J. Manthi**2**, Damaris A. Odeny**2**,**  **Charles A.O. Midega**1**, Zeyaur R. Khan**1**, John A. Pickett**4**, and Toby J.A. Bruce**5,*

1International Centre of Insect Physiology and Ecology (ICIPE), P.O. Box 30772-00100, Nairobi, Kenya

2International Crops Research Institute for the Semi-Arid Tropics (ICRISAT) P.O. Box 39063–00623, Nairobi, Kenya

3International Institute of Tropical Agriculture (IITA), PMB-5320, Ibadan, Nigeria

4School of Chemistry, Cardiff University, Cardiff, CF10 3AT, UK

5School of Life Sciences, Keele University, Staffordshire, ST5 5BG, UK

*t.j.a.bruce@keele.ac.uk

+these authors contributed equally to this work

**Supplementary Table 1.** Complete list of olfactometer responses of *Cotesia sesamiae* wasps to all maize genotypes. Entries are ranked according to *P-*value * = Significant at *P* < 0.05. Details of significant genotypes (i.e. those exhibiting the egg-induced parasitoid attraction trait) are provided in Table 2 of the main text.

| **Entry code** | **Category** | **Name** | **Replications screened** | **Mean Time Spent (Minutes)** | | | ***P*-value** | ***F-*value** |
| --- | --- | --- | --- | --- | --- | --- | --- | --- |
|  |  |  |  | **Without eggs** | **With Eggs** | **Solvent Control** |  |  |
| B4_6E1 | Landrace | VENEZUELA648 | 10 | 2.28 | 4.39 | 2.12 | <0.0001* | 14.46 |
| B8_7E3 | Inbred line | CML312 | 10 | 2.66 | 4.06 | 2.13 | <0.0001* | 16.60 |
| B8_8E6 | Inbred line | CML442 | 10 | 1.84 | 4.13 | 2.68 | <0.0001* | 21.84 |
| B14_9E3 | Inbred line | CKSPL10089 | 10 | 2.39 | 3.69 | 2.17 | 0.0001* | 11.96 |
| B14_12E3 | Inbred line | CKSPL10090 | 10 | 2.02 | 3.70 | 2.27 | 0.0001* | 10.63 |
| B16_8E1 | Inbred line | M211 | 13 | 2.76 | 4.00 | 2.15 | 0.0001* | 11.25 |
| B17_5E1 | Inbred line | MSMP-ZEBRA | 10 | 2.37 | 3.53 | 2.28 | 0.0001* | 10.79 |
| B11_1E4 | Inbred line | P300C5S1B | 10 | 2.54 | 3.87 | 2.40 | 0.0002* | 10.69 |
| B11_3E1 | Inbred line | P100C6-200 | 10 | 2.51 | 3.53 | 2.58 | 0.0002* | 10.09 |
| B14_1E2 | Inbred line | CKSPL10036 | 10 | 2.42 | 3.82 | 2.19 | 0.0002* | 10.07 |
| B16_2E1 | Hybrid | MASENO-EH-11 | 12 | 2.31 | 4.07 | 2.30 | 0.0002* | 10.13 |
| B16_4E1 | Inbred line | EX614-PX389 | 10 | 2.17 | 3.57 | 2.70 | 0.0003* | 10.35 |
| B2_1E5 | Hybrid | CKIR12001 | 10 | 2.01 | 4.25 | 2.25 | 0.0005* | 9.47 |
| B16_1E1 | Inbred line | X614-3 | 11 | 2.42 | 3.75 | 2.34 | 0.0005* | 9.18 |
| B13_1E1 | Inbred line | CKSBL10042 | 10 | 1.77 | 3.65 | 3.05 | 0.0006* | 9.18 |
| B13_4E1 | Inbred line | CKSBL10060 | 10 | 2.14 | 3.70 | 2.78 | 0.001* | 8.21 |
| B8_1E2 | Landrace | CUBA91 | 10 | 1.65 | 3.42 | 2.59 | 0.0012* | 8.06 |
| B8_5E6 | Landrace | JOWI-RED | 10 | 2.61 | 3.52 | 2.52 | 0.0012* | 7.79 |
| B8_9E13 | Inbred line | CKSBL10027 | 10 | 2.40 | 3.98 | 2.47 | 0.0017* | 7.59 |
| B14_3E3 | Inbred line | CKSPL10074 | 10 | 2.18 | 3.57 | 2.49 | 0.0017* | 7.59 |
| B18_2E1 | Hybrid | X87/02/312 F4-5 | 10 | 2.24 | 3.65 | 2.45 | 0.0019* | 7.47 |
| B13_11E2 | Inbred line | CKSPL10007 | 10 | 2.30 | 3.42 | 2.30 | 0.0021* | 7.03 |
| B4_5E6 | Landrace | NYAMULA | 12 | 2.42 | 3.55 | 2.61 | 0.0024* | 6.93 |
| B12_9E1 | Inbred line | CKSBL-10038 | 10 | 2.71 | 3.89 | 2.29 | 0.0024* | 6.75 |
| B11_2E8 | Inbred line | CKSBL-10001 | 10 | 2.60 | 3.40 | 2.55 | 0.0025* | 6.94 |
| B4_3E3 | Landrace | CUBA91 | 10 | 2.77 | 3.71 | 2.45 | 0.0028* | 7.07 |
| B8_6E6 | Landrace | HAITI24 | 10 | 2.28 | 3.62 | 2.29 | 0.0045* | 6.27 |
| B8_3E8 | Landrace | BRAZIL1006 | 10 | 2.44 | 3.54 | 2.87 | 0.0049* | 0.72 |
| B12_1E2 | Inbred line | CKSBL-10015 | 10 | 2.72 | 3.68 | 2.50 | 0.0056* | 5.91 |
| B12_8E2 | Inbred line | CKSBL-10035 | 10 | 2.47 | 4.04 | 2.49 | 0.007* | 5.61 |
| B12_7E1 | Inbred line | CKSBL-10034 | 10 | 2.35 | 3.62 | 2.70 | 0.0089* | 5.31 |
| B8_2E7 | Landrace | VENEZUELA648 | 10 | 1.72 | 3.47 | 2.71 | 0.009* | 5.37 |
| B15_2E1 | Inbred line | CKSPL10136 | 10 | 2.09 | 3.48 | 2.43 | 0.0103* | 5.19 |
| B18_18E1 | Inbred line | EX614X389 F3-1 | 10 | 2.55 | 3.22 | 2.39 | 0.0119* | 5.00 |
| B14_2E2 | Inbred line | CKSPL10042 | 10 | 2.43 | 3.61 | 2.33 | 0.0122* | 4.79 |
| B15_10E1 | Inbred line | CKSPL10256 | 10 | 2.25 | 3.37 | 2.52 | 0.0125* | 4.88 |
| B16_7E1 | Inbred line | ABR | 10 | 2.10 | 3.17 | 2.94 | 0.0138* | 4.99 |
| B14_9E2 | Inbred line | CKSPL10089 | 10 | 2.30 | 3.26 | 2.38 | 0.0145* | 4.82 |
| B13_3E1 | Inbred line | CKSBL10046 | 10 | 2.62 | 3.69 | 2.46 | 0.015* | 4.58 |
| B11_5E1 | Inbred line | CKSBL-10004 | 10 | 2.19 | 3.15 | 2.90 | 0.0164* | 4.67 |
| B13_2E4 | Inbred line | CKSBL10045 | 10 | 2.47 | 3.63 | 2.65 | 0.0226* | 4.21 |
| B7_7E2 | Inbred line | CML334 | 10 | 2.26 | 3.19 | 2.27 | 0.0328* | 3.75 |
| B12_5E1 | Inbred line | CKSBL-10033 | 10 | 2.16 | 3.74 | 2.76 | 0.0384* | 3.56 |
| B11_7E6 | Inbred line | CKSBL10003 | 10 | 2.47 | 3.49 | 2.71 | 0.04* | 2.97 |
| B18_1E4 | Inbred line | ET14 MASENO | 10 | 2.36 | 3.37 | 2.54 | 0.048* | 2.53 |
| B15_7E1 | Inbred line | CKSPL10212 | 10 | 2.85 | 2.93 | 2.28 | 0.0517 | 3.17 |
| B13_13E2 | Inbred line | CKSPL10035 | 10 | 3.16 | 2.91 | 2.22 | 0.0553 | 3.13 |
| B16_9E1 | Inbred line | F-WHITE | 10 | 1.87 | 2.86 | 3.02 | 0.0554 | 3.26 |
| B11_6E1 | Inbred line | CKSBL-10005 | 10 | 1.98 | 2.79 | 3.15 | 0.0566 | 3.70 |
| B16_12E1 | Inbred line | EX 614-F3-2 | 10 | 2.02 | 2.32 | 3.12 | 0.0584 | 3.14 |
| B17_3E2 | Inbred line | ABLEP | 10 | 2.21 | 2.53 | 2.83 | 0.0618 | 3.12 |
| B15_12E1 | Inbred line | CKSPL10309 | 10 | 2.44 | 2.95 | 2.88 | 0.0633 | 3.00 |
| B14_10E3 | Inbred line | CKSPL10087 | 10 | 2.68 | 3.10 | 2.39 | 0.0670 | 5.03 |
| B9_4E8 | Inbred line | CML488 | 10 | 2.42 | 2.51 | 2.94 | 0.0684 | 2.89 |
| B17_11E1 | Inbred line | EX87/02/312 F3-2-2 | 10 | 2.00 | 3.08 | 2.63 | 0.0739 | 2.85 |
| B15_10E2 | Inbred line | CKSPL10256 | 10 | 2.92 | 2.33 | 2.75 | 0.0779 | 1.69 |
| B4_1E5 | Landrace | SEFENSI | 12 | 1.97 | 2.65 | 3.07 | 0.0866 | 2.62 |
| B14_6E4 | Inbred line | CKSPL10080 | 10 | 2.48 | 3.02 | 2.45 | 0.0873 | 2.58 |
| B9_6E8 | Inbred line | CML511 | 10 | 1.96 | 2.48 | 2.49 | 0.0920 | 2.55 |
| B11_4E3 | Inbred line | CKSBL-10007 | 10 | 2.66 | 3.35 | 2.32 | 0.0981 | 7.02 |
| B15_12E4 | Inbred line | CKSPL10309 | 10 | 2.33 | 2.75 | 2.85 | 0.1042 | 2.41 |
| B2_8E2 | Hybrid | CKIR12010 | 10 | 3.14 | 3.12 | 2.49 | 0.1051 | 2.40 |
| B12_12E3 | Inbred line | CKSBL-10040 | 10 | 2.84 | 3.34 | 2.57 | 0.1053 | 2.38 |
| B7_6E6 | Inbred line | CML204 | 10 | 2.86 | 2.59 | 2.30 | 0.1094 | 2.35 |
| B9_2E4 | Inbred line | CML441 | 10 | 2.44 | 2.76 | 2.95 | 0.1135 | 2.31 |
| B15_11E1 | Inbred line | CKSPL10280 | 10 | 2.28 | 3.05 | 2.52 | 0.1216 | 2.25 |
| B15_1E2 | Inbred line | CKSPL10164 | 10 | 1.99 | 3.18 | 2.59 | 0.1222 | 2.24 |
| B17_10E1 | Inbred line | X87/02/312-8 | 10 | 2.15 | 2.74 | 2.72 | 0.1229 | 2.26 |
| B4_4E4 | Landrace | HAITI 24 | 10 | 2.3 | 4.01 | 2.44 | 0.1232 | 2.22 |
| B3_5E2 | Hybrid | CKIR12016 | 11 | 3.06 | 2.39 | 2.91 | 0.1239 | 2.21 |
| B18_3E1 | Inbred line | X87/02/312-F4-6 | 10 | 2.37 | 2.54 | 2.84 | 0.1275 | 2.21 |
| B14_8E1 | Inbred line | CKSPL10088 | 10 | 2.57 | 2.91 | 2.48 | 0.1346 | 2.10 |
| B17_7E1 | Inbred line | WEEVIL WHITE | 10 | 2.21 | 3.01 | 2.72 | 0.1402 | 2.09 |
| B2_6E4 | Hybrid | CKIR12008 | 10 | 3.16 | 2.58 | 2.59 | 0.1407 | 2.07 |
| B11_9E1 | Inbred line | CKSBL-10014 | 10 | 2.44 | 2.85 | 3.05 | 0.1434 | 2.05 |
| B15_6E4 | Inbred line | CKSPL10191 | 10 | 2.78 | 2.97 | 2.31 | 0.1457 | 2.01 |
| B13_10E4 | Inbred line | CKSPL10028 | 10 | 2.74 | 3.20 | 2.40 | 0.1511 | 1.00 |
| B18_14E1 | Inbred line | X87/02/312 F4-2DC | 10 | 2.55 | 3.18 | 2.48 | 0.1521 | 1.99 |
| B8_4E3 | Landrace | NYAMULA | 10 | 2.45 | 2.97 | 2.73 | 0.1612 | 1.92 |
| B9_9E3 | Inbred line | DTPWC9-F115 | 10 | 2.46 | 2.46 | 2.89 | 0.1727 | 1.84 |
| B2_4E1 | Hybrid | CKIR12006 | 12 | 2.44 | 3.21 | 2.87 | 0.1895 | 1.73 |
| B3_2E3 | Hybrid | CKIR12012 | 10 | 3.15 | 2.9 | 2.58 | 0.1980 | 1.69 |
| B4_7E5 | Landrace | ENDERE | 10 | 2.54 | 2.53 | 3.03 | 0.2286 | 1.54 |
| B16_3E1 | Inbred line | MSMP-ZEBRA (RECIPROCAL) | 11 | 2.43 | 2.94 | 2.84 | 0.2348 | 1.50 |
| B3_7E1 | Hybrid | CKIR12018 | 10 | 2.24 | 3.33 | 2.63 | 0.2366 | 1.50 |
| B13_6E1 | Inbred line | CKSBL10013 | 10 | 3.06 | 3.16 | 2.59 | 0.2395 | 1.48 |
| B3_3E6 | Hybrid | CKIR12013 | 10 | 2.56 | 3.29 | 2.73 | 0.2425 | 1.47 |
| B18_16E1 | Inbred line | X44/442 (SWEET CORN) | 10 | 2.43 | 2.03 | 2.74 | 0.2510 | 1.45 |
| B17_5E4 | Inbred line | MSMP-ZEBRA-2 | 10 | 2.33 | 3.14 | 2.56 | 0.2516 | 1.43 |
| B3_8E1 | Hybrid | CKIR12019 | 10 | 2.74 | 2.00 | 2.86 | 0.2717 | 1.35 |
| B3_4E4 | Hybrid | CKIR12014 | 10 | 2.68 | 3.11 | 2.66 | 0.2734 | 1.34 |
| B4_2E2 | Landrace | JOWI RED | 10 | 2.92 | 2.99 | 2.67 | 0.2894 | 1.28 |
| B2_1E4 | Hybrid | CKIR12001 | 12 | 2.74 | 3.15 | 2.79 | 0.2901 | 1.27 |
| B7_2E7 | Inbred line | CML144 | 10 | 2.71 | 3.04 | 2.38 | 0.2931 | 1.72 |
| B18_4E1 | Inbred line | EX-HANANAG-4 | 10 | 2.21 | 2.95 | 2.66 | 0.2938 | 1.29 |
| B17_2E1 | Inbred line | EX-44/42-2 | 10 | 2.28 | 2.80 | 2.74 | 0.3250 | 1.16 |
| B18_5E1 | Inbred line | X87/02/312F3-3-1 | 10 | 2.68 | 2.28 | 2.79 | 0.3317 | 1.14 |
| B2_5E1 | Hybrid | CKIR12007 | 9 | 3.12 | 2.85 | 2.55 | 0.3387 | 1.12 |
| B18_10E1 | Inbred line | EX614PRW | 10 | 2.34 | 2.81 | 2.64 | 0.3395 | 1.12 |
| B7_9E2 | Inbred line | CML440 | 10 | 2.11 | 2.51 | 2.52 | 0.3398 | 1.11 |
| B15_4E1 | Inbred line | CKSPL10170 | 10 | 2.57 | 2.75 | 2.37 | 0.3420 | 1.09 |
| B16_5E1 | Hybrid | MASENO-EH-12 | 10 | 3.05 | 2.50 | 2.73 | 0.3494 | 1.08 |
| B7_3E8 | Inbred line | CML159 | 10 | 2.76 | 2.57 | 2.23 | 0.3577 | 1.06 |
| B14_13E4 | Inbred line | CKSPL10112 | 10 | 2.67 | 2.61 | 2.35 | 0.3595 | 1.01 |
| B2_3E2 | Hybrid | CKIR12004 | 10 | 2.75 | 2.4 | 2.78 | 0.3806 | 0.99 |
| B18_13E1 | Inbred line | ABR/ABLEP/ABR FS-20R | 10 | 2.37 | 3.01 | 2.43 | 0.3962 | 0.95 |
| B15_5E3 | Inbred line | CKSPL10186 | 10 | 2.96 | 2.58 | 2.57 | 0.3967 | 0.95 |
| B2_2E1 | Hybrid | CKIR12003 | 10 | 2.81 | 2.36 | 3.09 | 0.4014 | 0.94 |
| B17_6E4 | Inbred line | MSVTOL-2 | 10 | 2.05 | 2.93 | 2.79 | 0.4098 | 0.92 |
| B16_11E1 | Inbred line | EX-614-PSD | 11 | 2.65 | 2.41 | 2.92 | 0.4145 | 0.90 |
| B7_5E7 | Inbred line | CML202 | 13 | 2.37 | 2.47 | 2.78 | 0.4159 | 0.89 |
| B3_6E1 | Hybrid | CKIR12017 | 10 | 2.34 | 2.70 | 2.84 | 0.4192 | 0.89 |
| B12_11E3 | Inbred line | CKSBL-10039 | 10 | 3.11 | 3.13 | 2.63 | 0.4237 | 0.87 |
| B17_4E1 | Inbred line | EXT-STR-150 | 10 | 2.31 | 2.31 | 2.77 | 0.4369 | 0.85 |
| B15_13E1 | Inbred line | CKSPL10230 | 10 | 3.03 | 2.29 | 2.91 | 0.4549 | 0.81 |
| B13_12E2 | Inbred line | CKSPL10021 | 10 | 2.67 | 2.84 | 2.55 | 0.4673 | 0.77 |
| B3_7E6 | Hybrid | CKIR12018 | 10 | 2.45 | 2.73 | 2.75 | 0.4705 | 0.77 |
| B7_8E7 | Inbred line | CML395 | 10 | 2.54 | 2.22 | 2.91 | 0.4809 | 0.75 |
| B13_5E3 | Inbred line | CKSBL10043 | 10 | 2.60 | 3.09 | 2.88 | 0.4846 | 0.74 |
| B13_9E2 | Inbred line | CML444 | 10 | 2.26 | 2.74 | 2.46 | 0.5044 | 0.69 |
| B16_10E1 | Inbred line | EX-6-20R | 10 | 2.37 | 2.53 | 3.03 | 0.5117 | 0.68 |
| B15_5E1 | Inbred line | CKSPL10186 | 10 | 2.81 | 2.41 | 2.45 | 0.5133 | 0.68 |
| B18_15E1 | Inbred line | EX614x389 F3-1 WHITE | 10 | 2.61 | 2.88 | 2.45 | 0.5181 | 0.67 |
| B9_8E8 | Inbred line | DTPWC9-F104 | 10 | 2.75 | 2.70 | 2.52 | 0.5194 | 0.67 |
| B9_10E8 | Inbred line | LPSC7-F64 | 10 | 2.78 | 2.89 | 2.67 | 0.5343 | 0.64 |
| B14_7E1 | Inbred line | CKSPL10085 | 10 | 2.90 | 3.03 | 2.20 | 0.5757 | 6.22 |
| B14_4E1 | Inbred line | CKSPL10070 | 10 | 2.47 | 3.22 | 2.57 | 0.5920 | 3.00 |
| B15_11E2 | Inbred line | CKSPL10280 | 10 | 2.62 | 2.87 | 2.69 | 0.5978 | 0.52 |
| B7_4E8 | Inbred line | CML197 | 11 | 2.50 | 2.75 | 2.73 | 0.6245 | 0.48 |
| B18_9E1 | Inbred line | X87/02/312 F3-2-1 | 10 | 2.42 | 2.64 | 2.65 | 0.6290 | 0.47 |
| B11_8E2 | Inbred line | CKSBL-10013 | 10 | 2.61 | 2.86 | 2.85 | 0.6374 | 0.46 |
| B9_3E4 | Inbred line | CML445 | 10 | 2.4 | 2.85 | 2.66 | 0.6429 | 0.45 |
| B12_6E4 | Inbred line | CKSBL-10028 | 10 | 2.64 | 2.75 | 3.05 | 0.6688 | 0.41 |
| B9_1E6 | Inbred line | CML443 | 10 | 2.52 | 2.93 | 2.81 | 0.6785 | 0.39 |
| B12_2E2 | Inbred line | CKSBL-10020 | 10 | 2.68 | 2.91 | 2.91 | 0.6829 | 0.39 |
| B17_9E1 | Inbred line | SAGAM | 10 | 2.66 | 2.79 | 2.59 | 0.6852 | 0.38 |
| B12_3E1 | Inbred line | CKSBL-10021 | 10 | 2.87 | 2.54 | 2.77 | 0.6865 | 0.38 |
| B15_6E1 | Inbred line | CKSPL10191 | 10 | 2.68 | 2.62 | 2.46 | 0.6889 | 0.38 |
| B12_10E1 | Inbred line | CKSBL-10041 | 10 | 2.86 | 3.07 | 2.71 | 0.6929 | 0.37 |
| B18_1E1 | Inbred line | X87/02/312 F4-5 | 10 | 2.53 | 2.77 | 2.67 | 0.7032 | 0.35 |
| B18_12E1 | Hybrid | EH10 MASENO | 10 | 2.30 | 2.64 | 2.68 | 0.7074 | 0.35 |
| B15_8E2 | Inbred line | CKSPL10273 | 10 | 2.64 | 2.74 | 2.56 | 0.7282 | 0.32 |
| B17_1E1 | Inbred line | EX-218 | 10 | 2.62 | 2.85 | 2.90 | 0.7341 | 0.31 |
| B15_3E4 | Inbred line | CKSPL10146 | 10 | 2.86 | 3.13 | 2.14 | 0.7419 | 3.45 |
| B2_7E1 | Hybrid | CKIR12009 | 10 | 2.87 | 3.00 | 2.71 | 0.7487 | 0.29 |
| B14_5E4 | Inbred line | CKSPL10081 | 10 | 2.60 | 2.81 | 2.73 | 0.7540 | 0.28 |
| B18_6E1 | Inbred line | WEEVIL PURPLE | 10 | 2.23 | 2.19 | 2.83 | 0.7620 | 3.54 |
| B14_11E1 | Inbred line | CKSPL10086 | 10 | 2.67 | 2.78 | 2.59 | 0.7797 | 0.25 |
| B9_5E1 | Inbred line | CZL01005 | 10 | 2.76 | 2.73 | 2.88 | 0.7917 | 0.24 |
| B9_7E2 | Inbred line | CZL03007 | 10 | 2.31 | 2.66 | 2.51 | 0.8093 | 0.21 |
| B4_1E1 | Landrace | SEFENSI | 10 | 2.65 | 2.69 | 2.85 | 0.8208 | 0.20 |
| B4_8E2 | Landrace | KONGERE | 12 | 2.79 | 2.40 | 2.63 | 0.8480 | 0.17 |
| B12_4E1 | Inbred line | CKSBL-10030 | 10 | 2.84 | 2.86 | 2.83 | 0.8497 | 0.16 |
| B18_7E1 | Inbred line | SAGAM EX87/102/312 F4 | 10 | 2.33 | 3.25 | 2.49 | 0.8530 | 2.60 |
| B17_8E1 | Inbred line | EX-YELLOW | 10 | 2.55 | 2.61 | 2.67 | 0.8675 | 0.14 |
| B3_1E5 | Hybrid | CKIR12011 | 10 | 2.8 | 3.04 | 2.89 | 0.8758 | 0.13 |
| B17_3E1 | Inbred line | ABLEP | 10 | 2.52 | 2.71 | 2.63 | 0.8994 | 0.11 |
| B18_8E1 | Inbred line | X87/02/312 F4-4 | 10 | 2.51 | 2.57 | 2.73 | 0.9175 | O.09 |
| B17_12E1 | Inbred line | EX87/312/ F4-1 | 10 | 2.68 | 2.70 | 2.55 | 0.9363 | 0.07 |
| B13_14E2 | Hybrid | SC-DUMA 43 | 10 | 2.77 | 2.54 | 2.54 | 0.9438 | 0.06 |
| B16_6E1 | Inbred line | 601-STR | 13 | 2.57 | 2.85 | 2.78 | 0.9472 | 0.05 |
| B15_9E3 | Inbred line | CKSPL10341 | 10 | 2.58 | 2.72 | 2.66 | 0.9482 | 0.05 |
| B13_7E1 | Inbred line | CKSBL10004 | 10 | 2.60 | 2.60 | 2.45 | 0.9495 | 0.05 |
| B18_11E1 | Inbred line | M7P | 10 | 2.54 | 2.60 | 2.58 | 0.9541 | 0.05 |
| B13_10E1 | Inbred line | CKSPL10028 | 10 | 2.63 | 2.66 | 2.58 | 0.9588 | 0.04 |
| B7_1E8 | Inbred line | CML78 | 10 | 2.62 | 2.39 | 2.68 | 0.9678 | 0.03 |
| B17_4E4 | Inbred line | EXT-STR-150 | 10 | 2.46 | 2.52 | 2.69 | 0.9681 | 0.03 |
| B13_8E1 | Inbred line | CML395 | 10 | 2.62 | 2.85 | 2.82 | 0.9800 | 0.02 |
| B15_8E1 | Inbred line | CKSPL10273 | 10 | 2.89 | 2.92 | 2.22 | 0.9876 | 5.33 |

**Supplementary Table 2.** Distribution and frequency of SNPs over the maize chromosomes for the GBS approach used.

| **Chromosome** | **Physical position (start and end)** | **Length covered (bp)** | **Number of SNPs** | **Kbs/SNP** | **SNP/mb** |
| --- | --- | --- | --- | --- | --- |
| 1 | 9776 - 3012522888 | 301242512 | 9275 | 32.48 | 30.79 |
| 2 | 10267 - 236966521 | 236956254 | 6818 | 34.75 | 28.77 |
| 3 | 1260616 - 193580216 | 192319600 | 6105 | 31.50 | 31.74 |
| 4 | 30838 - 241274836 | 241243998 | 4915 | 49.08 | 20.37 |
| 5 | 313 - 217019347 | 217019034 | 5930 | 36.60 | 27.32 |
| 6 | 129643 -169102723 | 168973080 | 4369 | 38.68 | 25.86 |
| 7 | 178709 - 176524193 | 176345484 | 4623 | 38.15 | 26.22 |
| 8 | 173438 - 175365170 | 175191732 | 4522 | 38.74 | 25.81 |
| 9 | 318633 - 156421953 | 156103320 | 4006 | 38.97 | 25.66 |
| 10 | 1280963 - 150107267 | 148826304 | 3748 | 39.71 | 25.18 |
| **Total** |  | **2014221318** | **54311** | **37.09** | **26.96** |

**Supplementary Table 3.** List of 101 SNPs significantly associated with the parasitoid attraction trait using GLM and MLM approaches. Entries are ranked according to position on the chromosome.

| **SNP No.** | **Chromosome** | **SNP position** | **GLM + PCA** | | | **MLM + PCA +K** | | |
| --- | --- | --- | --- | --- | --- | --- | --- | --- |
|  |  |  | ***P*-value** | **FDR adjusted *P*-value** | **Marker-R^2^** | ***P*-value** | **FDR adjusted *P*-value** | **Marker-R^2^** |
| 1 | 1 | S1_2793933 | 7.39E-07 | 0.0073444 | 0.19578 | 2.96E-05 | 0.02798717 | 0.15333 |
| 2 | 1 | S1_8430345 | 1.47E-11 | 0.0073444 | 0.27878 | 2.37E-07 | 0.02105182 | 0.21285 |
| 3 | 1 | S1_23589496 | 1.08E-09 | 0.0073444 | 0.30348 | 6.38E-06 | 0.02105182 | 0.22778 |
| 4 | 1 | S1_23589497 | 1.08E-09 | 0.0073444 | 0.30348 | 6.38E-06 | 0.02105182 | 0.22778 |
| 5 | 1 | S1_42807943 | 4.12E-09 | 0.0073444 | 0.24716 | 7.37E-06 | 0.02105182 | 0.18752 |
| 6 | 1 | S1_173266469 | 1.71E-11 | 0.0073444 | 0.29384 | 3.46E-07 | 0.02105182 | 0.22581 |
| 7 | 1 | S1_182904826 | 2.69E-07 | 0.0073444 | 0.17658 | 6.74E-06 | 0.02105182 | 0.15212 |
| 8 | 1 | S1_200500239 | 3.56E-18 | 0.0073444 | 0.49852 | 2.73E-09 | 0.02105182 | 0.40963 |
| 9 | 1 | S1_200860437 | 1.11E-04 | 0.01696204 | 0.13616 | 7.98E-06 | 0.02105182 | 0.17758 |
| 10 | 1 | S1_202298290 | 9.28E-13 | 0.0073444 | 0.35959 | 5.04E-08 | 0.02105182 | 0.26301 |
| 11 | 1 | S1_202445615 | 3.26E-09 | 0.0073444 | 0.28842 | 1.18E-05 | 0.02105182 | 0.29316 |
| 12 | 1 | S1_219232870 | 1.72E-07 | 0.0073444 | 0.20649 | 3.12E-05 | 0.02813699 | 0.15406 |
| 13 | 1 | S1_219573537 | 4.36E-08 | 0.0073444 | 0.24274 | 9.75E-06 | 0.02105182 | 0.18177 |
| 14 | 1 | S1_226222075 | 1.55E-12 | 0.0073444 | 0.32917 | 3.06E-07 | 0.02105182 | 0.24442 |
| 15 | 1 | S1_244559899 | 4.22E-10 | 0.0073444 | 0.30838 | 3.57E-07 | 0.02105182 | 0.22843 |
| 16 | 1 | S1_250301400 | 1.19E-12 | 0.0073444 | 0.32642 | 9.54E-08 | 0.02105182 | 0.2576 |
| 17 | 1 | S1_258047667 | 1.56E-09 | 0.0073444 | 0.24483 | 2.71E-07 | 0.02105182 | 0.20554 |
| 18 | 1 | S1_267568093 | 2.67E-04 | 0.03097607 | 0.12676 | 5.00E-05 | 0.03351832 | 0.14566 |
| 19 | 1 | S1_275027100 | 6.38E-14 | 0.0073444 | 0.406 | 1.28E-09 | 0.02105182 | 0.3657 |
| 20 | 1 | S1_289271440 | 2.63E-12 | 0.0073444 | 0.3542 | 8.63E-08 | 0.02105182 | 0.27977 |
| 21 | 1 | S1_291725718 | 0.00131 | 0.07791247 | 0.1098 | 3.81E-05 | 0.0302099 | 0.14687 |
| 22 | 2 | S2_29054622 | 5.14E-07 | 0.0073444 | 0.21707 | 6.37E-05 | 0.03612126 | 0.16299 |
| 23 | 2 | S2_29054624 | 5.14E-07 | 0.0073444 | 0.21707 | 6.37E-05 | 0.03612126 | 0.16299 |
| 24 | 2 | S2_29054625 | 5.14E-07 | 0.0073444 | 0.21707 | 6.37E-05 | 0.03612126 | 0.16299 |
| 25 | 2 | S2_29054647 | 5.14E-07 | 0.0073444 | 0.21707 | 6.37E-05 | 0.03612126 | 0.16299 |
| 26 | 2 | S2_32755546 | 1.37E-10 | 0.0073444 | 0.30494 | 3.99E-06 | 0.02105182 | 0.26992 |
| 27 | 2 | S2_47604347 | 1.21E-05 | 0.0073444 | 0.14961 | 4.01E-05 | 0.03043625 | 0.1316 |
| 28 | 2 | S2_47604351 | 1.21E-05 | 0.0073444 | 0.14961 | 4.01E-05 | 0.03043625 | 0.1316 |
| 29 | 2 | S2_50644399 | 3.62E-05 | 0.00949559 | 0.13139 | 6.40E-05 | 0.03612126 | 0.13974 |
| 30 | 2 | S2_180834407 | 1.18E-08 | 0.0073444 | 0.24484 | 1.87E-06 | 0.02105182 | 0.21334 |
| 31 | 2 | S2_236814368 | 3.48E-07 | 0.0073444 | 0.2059 | 9.19E-05 | 0.04780135 | 0.21297 |
| 32 | 3 | S3_5447609 | 3.00E-12 | 0.0073444 | 0.30006 | 2.65E-07 | 0.02105182 | 0.22889 |
| 33 | 3 | S3_202720889 | 1.68E-09 | 0.0073444 | 0.27434 | 1.23E-06 | 0.02105182 | 0.22412 |
| 34 | 3 | S3_216989090 | 7.39E-08 | 0.0073444 | 0.20718 | 4.21E-05 | 0.03043625 | 0.15325 |
| 35 | 3 | S3_230441690 | 1.42E-13 | 0.0073444 | 0.34716 | 1.12E-09 | 0.02105182 | 0.29573 |
| 36 | 3 | S3_230441714 | 1.42E-13 | 0.0073444 | 0.34716 | 1.12E-09 | 0.02105182 | 0.29573 |
| 37 | 4 | S4_11266704 | 2.47E-06 | 0.0073444 | 0.15182 | 7.09E-05 | 0.03880344 | 0.13575 |
| 38 | 4 | S4_28116948 | 5.29E-08 | 0.0073444 | 0.23675 | 2.89E-05 | 0.02798717 | 0.17633 |
| 39 | 4 | S4_63093023 | 1.86E-06 | 0.0073444 | 0.18765 | 1.15E-05 | 0.02105182 | 0.15942 |
| 40 | 4 | S4_63093026 | 1.86E-06 | 0.0073444 | 0.18765 | 1.15E-05 | 0.02105182 | 0.15942 |
| 41 | 4 | S4_63093029 | 1.86E-06 | 0.0073444 | 0.18765 | 1.15E-05 | 0.02105182 | 0.15942 |
| 42 | 4 | S4_63093042 | 1.86E-06 | 0.0073444 | 0.18765 | 1.15E-05 | 0.02105182 | 0.15942 |
| 43 | 4 | S4_63093047 | 1.86E-06 | 0.0073444 | 0.18765 | 1.15E-05 | 0.02105182 | 0.15942 |
| 44 | 4 | S4_170027069 | 5.34E-12 | 0.0073444 | 0.32338 | 2.61E-07 | 0.02105182 | 0.24079 |
| 45 | 4 | S4_183448288 | 1.80E-05 | 0.00781135 | 0.13509 | 7.70E-05 | 0.04103917 | 0.1416 |
| 46 | 4 | S4_233851115 | 2.19E-06 | 0.0073444 | 0.14964 | 6.04E-05 | 0.03612126 | 0.13469 |
| 47 | 5 | S5_13136976 | 4.16E-04 | 0.0409437 | 0.1094 | 3.52E-05 | 0.02900266 | 0.15538 |
| 48 | 5 | S5_18572100 | 1.87E-08 | 0.0073444 | 0.25076 | 5.06E-06 | 0.02105182 | 0.22452 |
| 49 | 5 | S5_18572101 | 1.87E-08 | 0.0073444 | 0.25076 | 5.06E-06 | 0.02105182 | 0.22452 |
| 50 | 5 | S5_18572107 | 1.87E-08 | 0.0073444 | 0.25076 | 5.06E-06 | 0.02105182 | 0.22452 |
| 51 | 5 | S5_18572110 | 1.87E-08 | 0.0073444 | 0.25076 | 5.06E-06 | 0.02105182 | 0.22452 |
| 52 | 5 | S5_18572112 | 1.87E-08 | 0.0073444 | 0.25076 | 5.06E-06 | 0.02105182 | 0.22452 |
| 53 | 5 | S5_18572113 | 1.87E-08 | 0.0073444 | 0.25076 | 5.06E-06 | 0.02105182 | 0.22452 |
| 54 | 5 | S5_80439617 | 6.52E-08 | 0.0073444 | 0.22545 | 7.92E-06 | 0.02105182 | 0.15056 |
| 55 | 5 | S5_205562793 | 8.98E-09 | 0.0073444 | 0.21072 | 2.97E-05 | 0.02798717 | 0.13167 |
| 56 | 5 | S5_205562798 | 8.98E-09 | 0.0073444 | 0.21072 | 2.97E-05 | 0.02798717 | 0.13167 |
| 57 | 5 | S5_206316844 | 5.13E-09 | 0.0073444 | 0.24603 | 3.51E-05 | 0.02900266 | 0.15918 |
| 58 | 5 | S5_210304624 | 2.85E-11 | 0.0073444 | 0.3504 | 7.00E-06 | 0.02105182 | 0.21722 |
| 59 | 6 | S6_28148054 | 7.83E-08 | 0.0073444 | 0.19428 | 3.94E-06 | 0.02105182 | 0.16097 |
| 60 | 6 | S6_84376158 | 5.65E-14 | 0.0073444 | 0.36081 | 2.65E-07 | 0.02105182 | 0.27014 |
| 61 | 6 | S6_84376161 | 5.65E-14 | 0.0073444 | 0.36081 | 2.65E-07 | 0.02105182 | 0.27014 |
| 62 | 6 | S6_122247887 | 2.90E-06 | 0.0073444 | 0.18078 | 3.95E-06 | 0.02105182 | 0.22075 |
| 63 | 6 | S6_122394808 | 1.53E-07 | 0.0073444 | 0.24362 | 5.28E-05 | 0.03422046 | 0.14956 |
| 64 | 6 | S6_122394809 | 1.53E-07 | 0.0073444 | 0.24362 | 5.28E-05 | 0.03422046 | 0.14956 |
| 65 | 7 | S7_135523090 | 1.45E-09 | 0.0073444 | 0.2859 | 1.34E-05 | 0.02137554 | 0.22913 |
| 66 | 7 | S7_147575520 | 9.01E-15 | 0.0073444 | 0.41918 | 2.11E-09 | 0.02105182 | 0.35757 |
| 67 | 7 | S7_164199102 | 1.20E-05 | 0.0073444 | 0.17421 | 6.18E-06 | 0.02105182 | 0.17906 |
| 68 | 7 | S7_174504226 | 1.65E-04 | 0.02289072 | 0.10863 | 6.20E-05 | 0.03612126 | 0.12307 |
| 69 | 7 | S7_175279256 | 1.39E-05 | 0.0074147 | 0.17554 | 4.69E-05 | 0.0322425 | 0.17553 |
| 70 | 8 | S8_825268 | 3.19E-04 | 0.03400575 | 0.11088 | 5.69E-05 | 0.03560174 | 0.14139 |
| 71 | 8 | S8_3163970 | 6.63E-12 | 0.0073444 | 0.36848 | 3.54E-07 | 0.02105182 | 0.27955 |
| 72 | 8 | S8_3799096 | 4.10E-05 | 0.00979806 | 0.15377 | 1.21E-05 | 0.02105182 | 0.16764 |
| 73 | 8 | S8_7299712 | 2.72E-07 | 0.0073444 | 0.22502 | 5.52E-05 | 0.03510147 | 0.14962 |
| 74 | 8 | S8_22686526 | 1.20E-07 | 0.0073444 | 0.1992 | 3.49E-05 | 0.02900266 | 0.15856 |
| 75 | 8 | S8_90258880 | 1.42E-04 | 0.02033778 | 0.1408 | 4.35E-05 | 0.03080501 | 0.14966 |
| 76 | 8 | S8_138888278 | 2.11E-12 | 0.0073444 | 0.34187 | 1.91E-07 | 0.02105182 | 0.26444 |
| 77 | 8 | S8_157671586 | 4.16E-04 | 0.0409437 | 0.12712 | 7.48E-05 | 0.04035283 | 0.15008 |
| 78 | 8 | S8_172187113 | 4.08E-06 | 0.0073444 | 0.17647 | 7.07E-05 | 0.03880344 | 0.15285 |
| 79 | 8 | S8_172187126 | 1.35E-05 | 0.00739118 | 0.1609 | 2.98E-05 | 0.02798717 | 0.16785 |
| 80 | 9 | S9_24196104 | 1.01E-07 | 0.0073444 | 0.18346 | 8.24E-06 | 0.02105182 | 0.14937 |
| 81 | 9 | S9_113242669 | 9.66E-14 | 0.0073444 | 0.3814 | 3.49E-07 | 0.02105182 | 0.28367 |
| 82 | 9 | S9_114280243 | 4.28E-04 | 0.04146851 | 0.09977 | 3.15E-05 | 0.02813699 | 0.13513 |
| 83 | 9 | S9_116948141 | 0.00107 | 0.06876613 | 0.10713 | 4.18E-05 | 0.03043625 | 0.15115 |
| 84 | 9 | S9_116948148 | 0.00107 | 0.06876613 | 0.10713 | 4.18E-05 | 0.03043625 | 0.15115 |
| 85 | 9 | S9_130499581 | 2.33E-09 | 0.0073444 | 0.28813 | 4.46E-07 | 0.02105182 | 0.2153 |
| 86 | 9 | S9_152534623 | 1.09E-07 | 0.0073444 | 0.19374 | 6.86E-06 | 0.02105182 | 0.17526 |
| 87 | 10 | S10_80491988 | 1.78E-07 | 0.0073444 | 0.18878 | 1.46E-05 | 0.02164978 | 0.16754 |
| 88 | 10 | S10_114811982 | 1.15E-11 | 0.0073444 | 0.28996 | 1.76E-06 | 0.02105182 | 0.2164 |
| 89 | 10 | S10_116675865 | 1.11E-09 | 0.0073444 | 0.24543 | 4.42E-06 | 0.02105182 | 0.2349 |
| 90 | 10 | S10_123814124 | 3.52E-07 | 0.0073444 | 0.21093 | 9.23E-05 | 0.04780135 | 0.15432 |
| 91 | 10 | S10_126494378 | 5.39E-11 | 0.0073444 | 0.32567 | 5.27E-07 | 0.02105182 | 0.33411 |
| 92 | 10 | S10_127808418 | 2.41E-09 | 0.0073444 | 0.25725 | 1.18E-05 | 0.02105182 | 0.18677 |
| 93 | 10 | S10_132009667 | 1.31E-07 | 0.0073444 | 0.23077 | 6.23E-05 | 0.03612126 | 0.15814 |
| 94 | 10 | S10_140974149 | 1.60E-09 | 0.0073444 | 0.28564 | 5.17E-07 | 0.02105182 | 0.21686 |
| 95 | 10 | S10_140974151 | 1.60E-09 | 0.0073444 | 0.28564 | 5.17E-07 | 0.02105182 | 0.21686 |
| 96 | 10 | S10_140974163 | 1.48E-09 | 0.0073444 | 0.28656 | 5.03E-07 | 0.02105182 | 0.2173 |
| 97 | 10 | S10_140974237 | 6.71E-10 | 0.0073444 | 0.28846 | 3.75E-07 | 0.02105182 | 0.21624 |
| 98 | 10 | S10_140974238 | 6.04E-10 | 0.0073444 | 0.28967 | 3.32E-07 | 0.02105182 | 0.21823 |
| 99 | 10 | S10_140974247 | 6.04E-10 | 0.0073444 | 0.28967 | 3.32E-07 | 0.02105182 | 0.21823 |
| 100 | 10 | S10_140974253 | 3.75E-10 | 0.0073444 | 0.2951 | 2.03E-07 | 0.02105182 | 0.22639 |
| 101 | 10 | S10_140974256 | 6.04E-10 | 0.0073444 | 0.28967 | 3.32E-07 | 0.02105182 | 0.21823 |

**Supplementary Table 4.** Detailed information about 33 candidate genes identified within a 10mb region of linked SNPs in 10 chromosomes across the maize genome (B73 RefGen_v2 maize database). These candidate genes are genes already annotated with plant defence functions. Chr = chromosome.

| **Chr** | **SNP position** | **Gene symbol** | **Gene name** | **Synonyms (from maizeGDB)** | **Gene product** | **Gene position in B73 RefGen_v2 (Start to end)** | **Link address** |
| --- | --- | --- | --- | --- | --- | --- | --- |
| 1 | **182904826** | *bx9* | benzoxazinone synthesis9 | benzoxazinone synthesis9, bx9, gnp_QBL10f08, gpm524, PCO129924, pco129924(50), pza00484, rs131186109 | [UDP-glucose:2,4-dihydroxy-7-methoxy-2H-1,4-benzoxazin-3(4H)-one 2-D-glucosyltransferase](https://www.maizegdb.org/data_center/gene_product?id=9021865) | 180306606 to 180308510 | https://www.maizegdb.org/gene_center/gene/bx9 |
| 1 | **200500239** | *opr7* | 12-oxo-phytodienoic acid reductase7 | opr7, PCO088243(64); | [12-oxo-phytodienoic acid reductase](https://www.maizegdb.org/data_center/gene_product?id=9020762) | 207901911 to 207905956 | https://www.maizegdb.org/gene_center/gene/opr7 |
|  |  | *hm1* | Helminthosporium carbonum susceptibility1 | umc276a | [NADPH HC-toxin reductase](https://www.maizegdb.org/data_center/gene_product?id=25551) | 199,754,467 to 199,756,329 | https://www.maizegdb.org/gene_center/gene?id=hm1 |
| 1 | **219232870** | *tps7* | terpene synthase7 | gnp_QCD28c06, gpm852, terpene synthase7, tps7 | [τ-cadinol synthase](https://www.maizegdb.org/data_center/gene_product?id=9035353) | 214886200 to 214892746 | https://www.maizegdb.org/gene_center/gene/tps7 |
| 1 | **23589496** | *aos2* | allene oxide synthesis2 | aos2, ZmAOS2a | [allene oxide synthase](https://www.maizegdb.org/data_center/gene_product?id=291381) | 28925827 to 28928044 | https://www.maizegdb.org/gene_center/gene/aos2 |
| 1 | **275027100** | *aos3* | allene oxide synthesis3 | aos3 | [allene oxide synthase](https://www.maizegdb.org/data_center/gene_product?id=291381) | 280850553 to 280852535 | https://www.maizegdb.org/gene_center/gene/aos3 |
| 1 | **289271440** | *bm2* | brown midrib2 | AY109096, bm2, brown midrib2, csu134a, csu134a(thf), methylenetetrahydrofolate reductase1, mtf1, PCO119715(100), ZmMTHFR-1 | [methylenetetrahydrofolate reductase](https://www.maizegdb.org/data_center/gene_product?id=954368) | 292089538 to 292095812 | https://www.maizegdb.org/gene_center/gene/bm2 |
| 3 | **202720889** | *fps3* | farnesyl diphosphate synthase3 | cl242_1(269), cl242_1b, fpps3, rs129403832 | [farnesyl diphosphate synthase](https://www.maizegdb.org/data_center/gene_product?id=9033906) | 205146239 to  205150759 | https://www.maizegdb.org/gene_center/gene/fps3 |
| 3 | **216989090** | *srph1* | SGT1 disease resistance protein homolo | IDP1602, PCO098564, PCO098564(275), SGT1, suppressor of G2 allele of skp1 | None; interacts with RACK1 protein involved in disease resistance | 217464401 to 217471380 | https://www.maizegdb.org/gene_center/gene/srph1 |
| 3 | **230441690** | *pme31* | pectin methylesterase31 | cl55825_1(280), cl55825_1b, putative pectinesterase 67 | [pectinesterase](https://www.maizegdb.org/data_center/gene_product?id=219506) | 228,719,170  to 228,720,718 | https://www.maizegdb.org/gene_center/gene?id=pme31 |
| 4 | **11266704** | *cystatin9* | psei9 (cystatin9) | CC9, cysteine proteinase inhibitor 8 | [cystatin, cysteine proteinase inhibitor](https://www.maizegdb.org/data_center/gene_product?id=975746) | 15320883  to 15321409 | https://www.maizegdb.org/gene_center/gene/cystatin9 |
| 4 | **28116948** | *bx7* | benzoxazinone synthesis7 | AY106107, benzoxazinone synthesis7, bx7, IDP478, O-methyltransferase, PCO065952, PCO088803(361), PCO088803a, PZA00115, umc2039 | [TRIBOA-glc O methyl transferase](https://www.maizegdb.org/data_center/gene_product?id=1204241) | 18221972 to 18222862 | https://www.maizegdb.org/gene_center/gene/bx7 |
| 5 | **80439617** | *tps2* | terpene synthase2 | S-linalool synthase, terpene synthase2, tps2 | [terpene synthase](https://www.maizegdb.org/data_center/gene_product?id=9022637) | 71263184 to 71266012 | https://www.maizegdb.org/gene_center/gene/tps2 |
|  |  | *tps3* | terpene synthase3 | S-linalool synthase, terpene synthase3, tps3 | [terpene synthase](https://www.maizegdb.org/data_center/gene_product?id=9022637) | 71380435 to 71383513 | https://www.maizegdb.org/gene_center/gene/tps3 |
| 6 | **84376158** | *mir1* | maize insect resistance1 | CL872_-2, maize insect resistance1, mir1, mir1(thp) | [cysteine protease](https://www.maizegdb.org/data_center/gene_product?id=25431) | 88909372 to 88911308 | https://www.maizegdb.org/gene_center/gene/mir1 |
|  |  | *mir2* | maize insect resistance2 | CL872_-2, gnp_AW438150, gpm228, maize insect resistance2, mir2, mir2(thp), thp*-Mp708, umc1178 | [cysteine protease--mir2](https://www.maizegdb.org/data_center/gene_product?id=113245) | 88903392 to 88908316 | https://www.maizegdb.org/gene_center/gene/mir2 |
|  |  | *chn2* | chitinase2 | Chit2, chitinase candidateL00973, chn*-L00973, pCh2, uiu5(chn) | [chitinase](https://www.maizegdb.org/data_center/gene_product?id=25130) | 82,862,563 to 82,863,789 | https://www.maizegdb.org/gene_center/gene?id=chn2 |
|  |  | *cdpk13* | calcium dependent protein kinase13 | cdpk*-D84508, ZmCRK3, calcium dependent protein kinase | [calcium dependent protein kinase](https://www.maizegdb.org/data_center/gene_product?id=56895) | 84,376,922 to 84,383,629 | https://www.maizegdb.org/gene_center/gene?id=cdpk13 |
|  |  | *mpk15* | MAP kinase15 | gnp_QCH4c06, gpm877, putative mitogen-activated protein kinase 17-3, MAP kinase (mitogen-activated protein kinase) | [MAP kinase](https://www.maizegdb.org/data_center/gene_product?id=223021) | 86,113,653 to 86,118,400 | https://www.maizegdb.org/gene_center/gene?id=mpk15 |
| 6 | **122394808** | *px5* | peroxidase 5 | gnp_AW461049, gpm233, PCO080642 | [peroxidase](https://www.maizegdb.org/data_center/gene_product?id=13862) | 125154676 to 125157008 | https://www.maizegdb.org/gene_center/gene/px5 |
| 7 | **147575520** | *ccp4* | cysteine protease4 | cathepsin B-like cysteine proteinase, ccp4(574), cl31697_1 | [cysteine protease](https://www.maizegdb.org/data_center/gene_product?id=25431) | 152772009 to 152776227 | https://www.maizegdb.org/gene_center/gene/ccp4 |
| 8 | **3163970** | *cystatin4* | psei4 (cystatin4) | cc4, CC4, cystatin3, multidomain cystatin | [cystatin, cysteine proteinase inhibitor](https://www.maizegdb.org/data_center/gene_product?id=975746) | 3347680 to 3350552 | https://www.maizegdb.org/gene_center/gene/cystatin4 |
| 8 | **138888278** | *mmt1* | methionine S-methyltransferase1 | cl1511_1, mmt1, PZA02033, S-adenosyl-L-methionine:L-methionine S-methyltransferase, methionine S-methyltransferase | [methionine S-methyltransferase](https://www.maizegdb.org/data_center/gene_product?id=923859) | 135,054,057 to 135,061,548 | https://www.maizegdb.org/gene_center/gene?id=mmt1 |
| 8 | **157671586** | *cystatin10* | psei10 (cystatin10) | cc10, CC10, cysteine proteinase inhibitor B | [cystatin, cysteine proteinase inhibitor](https://www.maizegdb.org/data_center/gene_product?id=975746) | 162579348  to 162580103 | https://www.maizegdb.org/gene_center/gene/cystatin10 |
| 8 | **172187113** | *cystatin2* | psei2 (cystatin2) | cc2, CCII, CC-II, csu223a(psei) , csu96, csu96b(psei) , cystatin II, gCC42, gnp_QAY2e12a, gpm416a, IDP119, ZmCyst (CK371502) | [cysteine proteinase inhibitor II](https://www.maizegdb.org/data_center/gene_product?id=109719) | 171412337  to 171415029 | https://www.maizegdb.org/gene_center/gene/cystatin2 |
| 9 | **152534623** | *aos1* | allene oxide synthase1 | aos1, IDP493, PCO060855, PCO060855(700), ZmAOS2b | [allene oxide synthase](https://www.maizegdb.org/data_center/gene_product?id=291381) | 145346839 to 145349218 | https://www.maizegdb.org/gene_center/gene/aos1 |
|  |  | *GRMZM2G438840 (0.27mb)* | MDIS1-interacting receptor like kinase 1 | LRR-RLK, LOC103639559 | Lucine-rich-repeat receptor kinase | 152256777 to 152260287 | https://www.maizegdb.org/gene_center/gene/GRMZM2G438840 |
| 10 | **80491988** | *tps10* | terpene synthase10 | sesquiterpene synthase10, terpene synthase10, tps10 | [sequiterpene synthase10](https://www.maizegdb.org/data_center/gene_product?id=1219901) | 74476281 to 74479851 | https://www.maizegdb.org/gene_center/gene/1219899 |
|  |  | *tps4* | terpene synthase4 | sesquiterpene synthase4, terpene synthase4, tps4 | [sesquiterpene synthase 4/5](https://www.maizegdb.org/data_center/gene_product?id=1219892) | 74010475 to 74013611 | https://www.maizegdb.org/gene_center/gene/1219891 |
|  |  | *tps5* | terpene synthase5 | sesquiterpene synthase5, terpene synthase5, tps5, TPS5 inactive sesquithujene synthase-like | [sesquiterpene synthase 4/5](https://www.maizegdb.org/data_center/gene_product?id=1219892) | 74236153 to 74239416 | https://www.maizegdb.org/gene_center/gene/1219895 |
|  |  | *tps9* | terpene synthase9 | IDP70, PCO101634b, terpene synthase9, tps9 | [sesquiterpene synthase 4/5](https://www.maizegdb.org/data_center/gene_product?id=1219892) | 73837673 to 73839016 | https://www.maizegdb.org/gene_center/gene/tps9 |
| 10 | **116675865** | *scp1* | serine carboxypeptidase1 | pco137899, scp1, ZmSCP may play at role in the disease resistance response | [serine carboxypeptidase](https://www.maizegdb.org/data_center/gene_product?id=9024124) | 120278216 to 120282881 | https://www.maizegdb.org/gene_center/gene/tps10 |
| 10 | **126494378** | *pme1* | pectin methylesterase1 | CL1790_1, CL1790_1(750), ZmPME7, pectinesterase | [pectinesterase](https://www.maizegdb.org/data_center/gene_product?id=219506) | 122,230,413 to 122,232,602 | https://www.maizegdb.org/gene_center/gene?id=pme1 |

**Supplementary Table 5.** Detailed information about 202 genes found within a 10mb region of the top 16 SNPs (selected based on having an R^2^ value of above 25% (0.25) in MLM approach) in 10 chromosomes across the maize genome (B73 RefGen_v2 maize database). Chr = chromosome.

| **SNP No.** | **SNP position** | **Gene name** | **Gene model name in maize database** | **Distance from SNP (mb)** | **Synonyms (from maizeGDB)** | **Gene product** | **Gene position in B73 RefGen_v2 (Start to end)** | **Link address** |
| --- | --- | --- | --- | --- | --- | --- | --- | --- |
| **1** | **Chr1_200500239** | [col16 - C2C2-CO-like-transcription factor 16](https://www.maizegdb.org/gene_center/gene/col16) | GRMZM2G159996 | 4.94 | BBX13, constans-like16, PZA00619, rs131832121, umc1709, ZmBBX2, ZmCOL03, ZmOrphan268 | B-box, CCT domain protein | 195,557,037 to 195,561,667 | <https://www.maizegdb.org/gene_center/gene?id=GRMZM2G159996> |
|  |  | [bhlh173 - bHLH-transcription factor 173](https://www.maizegdb.org/gene_center/gene/bhlh173) | GRMZM5G882527 | 4.78 | si683023f08, si683023f08(59), Transcription factor bHLH137 | None | 195,720,311 to 195,722,763 | <https://www.maizegdb.org/gene_center/gene?id=GRMZM5G882527> |
|  |  | [Zm00001d031666](https://www.maizegdb.org/gene_center/gene/Zm00001d031666) | GRMZM2G020631 | 4.75 | [In2-2, putative oxidoreductase, aldo/keto reductase family protein](https://www.maizegdb.org/person/59795) | None | 195,752,951 to 195,756,226 | <https://www.maizegdb.org/gene_center/gene?id=GRMZM2G020631> |
|  |  | [dsc2 - Discolored-paralog2](https://www.maizegdb.org/gene_center/gene/dsc2) | GRMZM5G872204 | 4.74 | putative ARF GTPase activating domain protein with ankyrin repeat-containing protein | [ADP-ribosylation factor GTPase activating protein](https://www.maizegdb.org/data_center/gene_product?id=9022708) | 195,758,423 to 195,770,006 | <https://www.maizegdb.org/gene_center/gene?id=GRMZM5G872204> |
|  |  | [cl765_1](https://www.maizegdb.org/gene_center/gene/cl765_1) | GRMZM2G452523 | 4.24 | [Biotin synthase, cl765_1](https://www.maizegdb.org/person/59795) | None | 196,263,561 to 196,267,558 | <https://www.maizegdb.org/gene_center/gene?id=GRMZM2G452523> |
|  |  | [bzip62 - bZIP-transcription factor 62](https://www.maizegdb.org/gene_center/gene/bzip62) | GRMZM2G332294 | 4.13 | None | None | 196,371,273 to 196,374,150 | <https://www.maizegdb.org/gene_center/gene?id=GRMZM2G332294> |
|  |  | [sodh1 - sorbitol dehydrogenase homolog1](https://www.maizegdb.org/gene_center/gene/sodh1) | GRMZM2G175423 | 6.07 | 05c04a01, PCO126824, sdh1, uaz152, uaz152(sdh), umc1499 | [sorbitol dehydrogenase](https://www.maizegdb.org/data_center/gene_product?id=66903) | 196,371,273 to 196,374,150 | <https://www.maizegdb.org/gene_center/gene?id=GRMZM2G175423> |
|  |  | [psa2 - photosystemI2](https://www.maizegdb.org/gene_center/gene/psa2) | GRMZM2G021687 | 4.45 | None | [protein disulfide isomerase](https://www.maizegdb.org/data_center/gene_product?id=86830) | 197,846,348 to 197,848,301 | <https://www.maizegdb.org/gene_center/gene?id=GRMZM2G021687> |
|  |  | GRMZM2G380227 | GRMZM2G380227 | 2.93 | [probable receptor-like protein kinase At5g15080-like, Serine/threonine-protein kinase (SIK: salt-inducible kinase)](https://www.maizegdb.org/person/59795) | None | 197,574,915 to 197,577,569 | <https://www.maizegdb.org/gene_center/gene?id=GRMZM2G380227> |
|  |  | [hb129 - Homeobox-transcription factor 129](https://www.maizegdb.org/gene_center/gene/hb129) | GRMZM2G142962 | 2.31 | homeobox-leucine zipper protein HOX27 | None | 198,192,712 to 198,194,680 | <https://www.maizegdb.org/gene_center/gene?id=GRMZM2G142962> |
|  |  | [zip1 - zinc-regulated, iron-regulated transporter-like protein1](https://www.maizegdb.org/gene_center/gene/zip1) | GRMZM2G001803 | 1.85 | Zinc transporter SLC39A7 | [zinc-regulated, iron-regulated transporter](https://www.maizegdb.org/data_center/gene_product?id=9041128) | 198,649,343 to 198,655,981 | <https://www.maizegdb.org/gene_center/gene?id=GRMZM2G001803> |
|  |  | [nnr4 - nitrate reductase4](https://www.maizegdb.org/gene_center/gene/nnr4) | GRMZM2G076723 | 1.76 | [nitrate reductase [NADH]-like, ZmNIAd](https://www.maizegdb.org/person/59795) | [nitrate reductase (NADH)](https://www.maizegdb.org/data_center/gene_product?id=25899) | 198,738,739 to 198,743,220 | <https://www.maizegdb.org/gene_center/gene?id=GRMZM2G076723#!> |
|  |  | [bzip49 - bZIP-transcription factor 49](https://www.maizegdb.org/gene_center/gene/bzip49) | GRMZM2G478417 | 1.02 | None | None | 199,484,975 to 199,491,206 | <https://www.maizegdb.org/gene_center/gene?id=GRMZM2G478417> |
|  |  | [hm1 - Helminthosporium carbonum susceptibility1](https://www.maizegdb.org/gene_center/gene/hm1) | GRMZM5G881887 | 0.75 | umc276a | [NADPH HC-toxin reductase](https://www.maizegdb.org/data_center/gene_product?id=25551) | 199,754,467 to 199,756,329 | <https://www.maizegdb.org/gene_center/gene?id=GRMZM5G881887> |
|  |  | cl12107_1 | GRMZM2G157010 | 0.03 | UPF0029 superfamily protein | None | 200,470,494 to 200,479,736 | <https://www.maizegdb.org/gene_center/gene?id=GRMZM2G157010> |
|  |  |  |  |  |  |  |  |  |
| **2** | **Chr1_202298290** | [cyc14 - cyclin14](https://www.maizegdb.org/gene_center/gene/cyc14) | GRMZM2G133413 | 1.57 | CycD4:2, cyclin-D4-2-like, rs128842854, umc2237 | [cyclin](https://www.maizegdb.org/data_center/gene_product?id=86017) | 200,723,661 to 200,726,940 | <https://www.maizegdb.org/gene_center/gene?id=GRMZM2G133413> |
|  |  | [tena2 - thiaminase2](https://www.maizegdb.org/gene_center/gene/tena2) | GRMZM2G148896 | 0.56 | Thiaminase II | TENA (also known as 4-amino-5-aminomethyl-2-methylpyrimidine aminohydrolase) | 201,736,178 to 201,740,436 | <https://www.maizegdb.org/gene_center/gene?id=GRMZM2G148896> |
|  |  | [ereb137 - AP2-EREBP-transcription factor 137](https://www.maizegdb.org/gene_center/gene/ereb137) | GRMZM2G028386 | 0.34 | ethylene-responsive transcription factor ABI4 | None | 201,957,089 to 201,958,672 | <https://www.maizegdb.org/gene_center/gene?id=GRMZM2G028386> |
|  |  | [br2 - brachytic2](https://www.maizegdb.org/gene_center/gene/br2) | GRMZM2G315375 | 0.001 | abcb1, Hahn 6 dwarf, mi1, Oakes dwarf, pgp1, qpa1, R4 dwarf, rs131839167 , umc2238, umc2239, ZmABCB4 | [BR2](https://www.maizegdb.org/data_center/gene_product?id=9022566) | 202,298,103 to 202,305,287 | <https://www.maizegdb.org/gene_center/gene?id=GRMZM2G315375> |
|  |  |  |  |  |  |  |  |  |
| **3** | **Chr1_202445615** | [pht7 - phosphate transporter protein7](https://www.maizegdb.org/gene_center/gene/pht7) | GRMZM2G112377 | 0.1 | Pht1;3, ZmPHT1;3, Zmpt3 | [phosphate transporter, plasma membrane](https://www.maizegdb.org/data_center/gene_product?id=9035041) | 202,549,102 to 202,551,276 | <https://www.maizegdb.org/gene_center/gene?id=GRMZM2G112377> |
|  |  | [hct12 - hydroxycinnamoyltransferase12](https://www.maizegdb.org/gene_center/gene/hct12) | GRMZM2G179703 | 0.47 | acyltransferase, ZmSLG | [hydroxycinnamoyltransferase](https://www.maizegdb.org/data_center/gene_product?id=9034276) | 202,919,151 to 202,921,697 | <https://www.maizegdb.org/gene_center/gene?id=GRMZM2G179703> |
|  |  | [nactf89 - NAC-transcription factor 89](https://www.maizegdb.org/gene_center/gene/nactf89) | GRMZM2G340305 | 0.64 | [nac89, NAC domain-containing protein 53](https://www.maizegdb.org/person/9021409) | None | 203,087,262 to 203,092,166 | <https://www.maizegdb.org/gene_center/gene?id=GRMZM2G340305> |
|  |  | [mdh6 - malate dehydrogenase6](https://www.maizegdb.org/gene_center/gene/mdh6) | GRMZM2G129513 | 0.72 | csu374b, gnp_QCK20f01, gpm886, NADP-MDH, PCO072788, rs128846139, rs128846153, rs131175317, rs131175318 i, rs131175319, umc1661 | [malate dehydrogenase (NADP+), chloroplast](https://www.maizegdb.org/data_center/gene_product?id=40092) | 203,170,067 to 203,174,226 | <https://www.maizegdb.org/gene_center/gene?id=GRMZM2G129513> |
|  |  | [zrp2 - Zea root protein2](https://www.maizegdb.org/gene_center/gene/zrp2) | GRMZM2G106980 | 1.2 | BURP3, BURP domain protein RD22, siU38791, ZmBURP07 ZmBURP08 | [BURP domain protein](https://www.maizegdb.org/data_center/gene_product?id=9037396) | 203,647,149 to 203,653,762 | <https://www.maizegdb.org/gene_center/gene?id=GRMZM2G106980> |
|  |  | [krp4 - kinesin-related protein4](https://www.maizegdb.org/gene_center/gene/krp4) | GRMZM2G320689 | 2.41 | [IDP687, kin4, KIN4, PCO120417b, php20855, ZmaKIN4](https://www.maizegdb.org/person/146822) | [kinesin](https://www.maizegdb.org/data_center/gene_product?id=276102) | 204,859,441 to 204,866,617 | <https://www.maizegdb.org/gene_center/gene?id=GRMZM2G320689> |
|  |  | [hp1 - histidine-containing phosphotransfer protein1](https://www.maizegdb.org/gene_center/gene/hp1) | GRMZM2G016439 | 2.77 | ZmHP1 | None | 205,219,468 to 205,222,558 | <https://www.maizegdb.org/gene_center/gene?id=GRMZM2G016439> |
|  |  | [iddp1 - indeterminate domain p1](https://www.maizegdb.org/gene_center/gene/iddp1) | GRMZM2G179677 | 3.67 | gnp_Id1b, gpm243b | [indeterminate domain protein](https://www.maizegdb.org/data_center/gene_product?id=952733) | 206,117,307 to 206,124,458 | <https://www.maizegdb.org/gene_center/gene?id=GRMZM2G179677> |
|  |  | umc1374 | GRMZM2G119761 | 4.07 | 26S proteasome non-ATPase regulatory subunit 3 homolog A, rs128852333 | None | 206,510,617 to 206,514,173 | https://www.maizegdb.org/gene_center/gene?id=GRMZM2G119761 |
|  |  | [srs1 - SHI/STY (SRS)-transcription factor 1](https://www.maizegdb.org/gene_center/gene/srs1) | GRMZM2G042407 | 4.94 | lateral root primordia like3, LRL3, SRS2 | [SHI/STY (SRS)-transcription factor](https://www.maizegdb.org/data_center/gene_product?id=9031348) | 207,389,667 to 207,399,144 | <https://www.maizegdb.org/gene_center/gene?id=GRMZM2G042407> |
|  |  |  |  |  |  |  |  |  |
| **4** | **Chr1_250301400** | [ptpn1 - protein tyrosine phosphatase-like nulceotidase1](https://www.maizegdb.org/gene_center/gene/ptpn1) | GRMZM2G146819 | 3.65 | PTP-like Phosphatase | [nucleotidase](https://www.maizegdb.org/data_center/gene_product?id=9043139) | 246,654,675 to 246,667,719 | <https://www.maizegdb.org/gene_center/gene?id=GRMZM2G146819> |
|  |  | [dof32 - C2C2-Dof-transcription factor 32](https://www.maizegdb.org/gene_center/gene/dof32) | GRMZM2G011832 | 3.82 | DOF5.7, ZmDof08 | None | 246,479,708 to 246,481,005 | <https://www.maizegdb.org/gene_center/gene?id=GRMZM2G011832> |
|  |  | [mterf3 - mTERF domain protein3](https://www.maizegdb.org/gene_center/gene/mterf3) | GRMZM2G034217 | 2.44 | Atmterf27, mTRF8 - Putative mitochondrial transcription termination factor family protein isoform 1, ZmTERF3 | None | 247,865,665 to 247,869,196 | <https://www.maizegdb.org/gene_center/gene?id=GRMZM2G034217> |
|  |  | [fha3 - FHA-transcription factor 3](https://www.maizegdb.org/gene_center/gene/fha3) | GRMZM2G086138 | 1.98 | None | None | 248,318,894 to 248,327,562 | <https://www.maizegdb.org/gene_center/gene?id=GRMZM2G086138> |
|  |  | ufg10 | GRMZM2G058057 | 0.89 | pco124850, pco124850(81), rhicadhesin receptor precursor | None | 249,414,646 to 249,417,825 | <https://www.maizegdb.org/gene_center/gene?id=GRMZM2G058057> |
|  |  | [ca2p6 - CCAAT-HAP2-transcription factor 26](https://www.maizegdb.org/gene_center/gene/ca2p6) | GRMZM5G829103 | 0.02 | nuclear transcription factor Y subunit A-10, rs128928950, umc1914, ZmNF-YA4 | [NF-YA, CCAAT-box binding protein subunit A](https://www.maizegdb.org/data_center/gene_product?id=9035478) | 250,317,114 to 250,321,412 | <https://www.maizegdb.org/gene_center/gene?id=GRMZM5G829103> |
|  |  | [myb73 - MYB-transcription factor 73](https://www.maizegdb.org/gene_center/gene/myb73) | GRMZM2G121570 | 2.02 | None | None | 252,320,123 to 252,322,173 | <https://www.maizegdb.org/gene_center/gene?id=GRMZM2G121570> |
|  |  | [bhlh43 - bHLH-transcription factor 43](https://www.maizegdb.org/gene_center/gene/bhlh43) | GRMZM2G165042 | 2.07 | bnl8.10a, Phytochrome-interacting factor, PIL5, putative HLH DNA-binding domain superfamily protein, ZmbHLH27, ZmPIF4.2, ZmPIF5 | None | 252,374,241 to 252,377,817 | <https://www.maizegdb.org/gene_center/gene?id=GRMZM2G165042> |
|  |  |  |  |  |  |  |  |  |
| **5** | **Chr1_275027100** | [tua1 - alpha tubulin1](https://www.maizegdb.org/gene_center/gene/tua1) | GRMZM2G153292 | 3.92 | bcd808, IDP2525, rs131176914, rs131892013, tub2, TubA2, Tubalpha2, tubulin alpha-2 chain-like | [alpha tubulin](https://www.maizegdb.org/data_center/gene_product?id=17141) | 271,106,011 to 271,114,338 | <https://www.maizegdb.org/gene_center/gene?id=GRMZM2G153292> |
|  |  | [kn1 - knotted1](https://www.maizegdb.org/gene_center/gene/kn1) | GRMZM2G017087 | 3.69 | AY107752, gsy19(kn1), homeobox protein OSH1, OSH1, PCO065671, sc19, SC19, ZmHB1 | None | 271,340,805 to 271,348,525 | <https://www.maizegdb.org/gene_center/gene?id=GRMZM2G017087> |
|  |  | [(emp4 - empty pericarp4)](https://www.maizegdb.org/gene_center/gene/emp4) | GRMZM2G092198 | 2.99 | aborted seed*-7065, abs*-7065, pentatricopeptide repeat protein-like, ZmPPR069 | [PPR EMP4 pentatricopeptide repeat protein; PPR pentatricopeptide repeat protein](https://www.maizegdb.org/data_center/gene_product?id=1233616) | 272,034,519 to 272,036,983 | <https://www.maizegdb.org/gene_center/gene?id=GRMZM2G092198> |
|  |  | [acs6 - 1-aminocyclopropane-1-carboxylate synthase6](https://www.maizegdb.org/gene_center/gene/acs6) | GRMZM2G054361 | 3.26 | ccs6, ACC synthase6, ACS65, ZmAcs6, ZmACS6 | [1-aminocyclopropane-1-carboxylate synthase](https://www.maizegdb.org/data_center/gene_product?id=953757) | 271,768,133 to 271,770,623 | <https://www.maizegdb.org/gene_center/gene?id=GRMZM2G054361> |
|  |  | grftf1 (GRF-transcription factor 1) | GRMZM2G178261 | 2.68 | [cl3497_1, grf1, Growth-regulating factor 2](https://www.maizegdb.org/person/9021409) | None | 272,348,922 to 272,353,057 | <https://www.maizegdb.org/gene_center/gene?id=GRMZM2G178261> |
|  |  | [cap4 - calcium pump4](https://www.maizegdb.org/gene_center/gene/cap4) | AC233878.1_FG004 | 2.38 | eca4, endoplasmic reticulum calcium ATPase2 | [calcium ATPase](https://www.maizegdb.org/data_center/gene_product?id=112964) | 272,645,068 to 272,663,927 | <https://www.maizegdb.org/gene_center/gene?id=AC233878.1_FG004> |
|  |  | [agpll1 - ADP glucose pyrophosphorylase large subunit leaf1AGPL4](https://www.maizegdb.org/gene_center/gene/agpll1) | GRMZM2G391936 | 1.58 | AGPL4, agpllzm, mmp83, plastid ADP-glucose pyrophosphorylase large subunit, ZmagpL1 | [ADP glucose pyrophosphorylase, leaf -- large subunit](https://www.maizegdb.org/data_center/gene_product?id=975548) | 273,447,466 to 273,452,300 | <https://www.maizegdb.org/gene_center/gene?id=GRMZM2G391936> |
|  |  | [aps1 - ATP sulfurylase1](https://www.maizegdb.org/gene_center/gene/aps1) | GRMZM2G149952 | 0.06 | ATP-sulfurylase 3, chloroplastic-like, bifunctional 3-phosphoadenosine 5-phosphosulfate synthetase, cl30_1d, rz630a, sed1, senescence-diminished1, ZmAS1 | [sulfate adenyltransferase](https://www.maizegdb.org/data_center/gene_product?id=113847) | 275,090,703 to 275,094,517 | <https://www.maizegdb.org/gene_center/gene?id=GRMZM2G149952> |
|  |  | [mgt5 - magnesium transporter5](https://www.maizegdb.org/gene_center/gene/mgt5) | GRMZM2G159295 | 0.04 | [pco092863, pco092863](https://www.maizegdb.org/person/136031) | None | 274,985,380 to 274,991,947 | <https://www.maizegdb.org/gene_center/gene?id=GRMZM2G159295> |
|  |  | [phd15 - PHD-transcription factor 15](https://www.maizegdb.org/gene_center/gene/phd15) | GRMZM2G173852 | 0.95 | Acyl-CoA N-acyltransferase with RING/FYVE/PHD-type zinc finger protein | None | 275,974,954 to 275,984,429 | <https://www.maizegdb.org/gene_center/gene?id=GRMZM2G173852> |
|  |  | [phyC1 - phytochromeC1](https://www.maizegdb.org/gene_center/gene/phyC1) | GRMZM2G057935 | 1.97 | phytochrome C1 | [phytochrome C](https://www.maizegdb.org/data_center/gene_product?id=978955) | 276,992,819 to 276,997,822 | <https://www.maizegdb.org/gene_center/gene?id=GRMZM2G057935> |
|  |  | [gsht1 - glutathione transporter1](https://www.maizegdb.org/gene_center/gene/gsht1) | GRMZM2G421491 | 1.79 | [oligopeptide transporter 3, ZmGT1](https://www.maizegdb.org/person/59795) | [glutathione transporter1](https://www.maizegdb.org/data_center/gene_product?id=9023824) | 276,815,648 to 276,820,449 | <https://www.maizegdb.org/gene_center/gene?id=GRMZM2G421491> |
|  |  | [mads4 - MADS-transcription factor 4](https://www.maizegdb.org/gene_center/gene/mads4) | GRMZM2G032339 | 2.12 | Agamous-like MADS-box protein AGL8 , m4 protein, ZMM4 | None | 277,151,350 to 277,232,124 | <https://www.maizegdb.org/gene_center/gene?id=GRMZM2G032339> |
|  |  |  |  |  |  |  |  |  |
| **6** | **Chr1_284271440** | [vps35 - vacuolar protein sorting35](https://www.maizegdb.org/gene_center/gene/vps35) | GRMZM5G825524 | 0.08 | ZmVPS35a | None | 289,192,462 to 289,217,741 | <https://www.maizegdb.org/gene_center/gene?id=GRMZM5G825524> |
|  |  | [bzip29 - bZIP-transcription factor 29](https://www.maizegdb.org/gene_center/gene/bzip29) | GRMZM2G138340 | 1.83 | DNA-binding protein EMBP-1, Embp-2 | None | 291,101,093 to 291,104,897 | <https://www.maizegdb.org/gene_center/gene?id=GRMZM2G138340> |
|  |  | pco110869 | GRMZM2G134227 | 1.98 | pco110869, putative protein phosphatase 2C 55 | None | 291,254,272 to 291,259,644 | <https://www.maizegdb.org/gene_center/gene?id=GRMZM2G134227> |
|  |  | [bm2 - brown midrib2](https://www.maizegdb.org/gene_center/gene/bm2) | GRMZM2G347056 | 2.82 | AY109096, csu134a, csu134a, csuh134a, methylenetetrahydrofolate reductase1, mtf1, PCO119715, umc372a, ZmMTHFR-1 | [methylenetetrahydrofolate reductase](https://www.maizegdb.org/data_center/gene_product?id=954368) | 292,089,538 to 292,095,812 | <https://www.maizegdb.org/gene_center/gene?id=GRMZM2G347056> |
|  |  | [nactf49 - NAC-transcription factor 49](https://www.maizegdb.org/gene_center/gene/nactf49) | GRMZM2G347043 | 2.82 | NAC1 transcription factor, nac49, ZmSNAC1 | NAC-type transcription factor | 292,086,460 to 292,088,246 | <https://www.maizegdb.org/gene_center/gene?id=GRMZM2G347043> |
|  |  | [alf15 - Alfin-like-transcription factor 15](https://www.maizegdb.org/gene_center/gene/alf15) | AC225147.4_FG003 | 3.6 | ZmAL1 | None | 292,875,307 to 292,879,433 | <https://www.maizegdb.org/gene_center/gene?id=AC225147.4_FG003> |
|  |  | [ts6 - tasselseed6](https://www.maizegdb.org/gene_center/gene/ts6) | GRMZM5G862109 | 3.62 | cl1812_1, ids1, indeterminate spikelet1, pge18, pzd00069, reversed germ orientation2, rgo2, rs128281361, rs131175377, ss196414883, ss196414885, tassel seed6, umc1009, ZmEREB11 | [Apetala2 transcription factor-like](https://www.maizegdb.org/data_center/gene_product?id=315221) | 292,889,740 to 292,893,983 | <https://www.maizegdb.org/gene_center/gene?id=GRMZM5G862109> |
|  |  | [adf3 - actin depolymerizing factor3](https://www.maizegdb.org/gene_center/gene/adf3) | GRMZM2G060702 | 3.89 | ABP3, ADF3, pco115125, pco115125a, umc84a, ZmABP3, ZmADF3 | [Actin depolymerizing factor](https://www.maizegdb.org/data_center/gene_product?id=113738) | 293,162,401 to 293,166,106 | <https://www.maizegdb.org/gene_center/gene?id=GRMZM2G060702> |
|  |  |  |  |  |  |  |  |  |
| **7** | **Chr1_27755546** | [sam2 - S-adenosyl methionine decarboxylase2](https://www.maizegdb.org/gene_center/gene/sam2) | GRMZM2G154397 | 0.26 | AY103590, csu6a, csu6a, csu6, gnp_QAW2d11a, gpm411a, gsy108, MZ2G1, PCO098412, sam*-mz6g1, sam*-Y07767, umc1326 | [S-adenosylmethionine decarboxylase](https://www.maizegdb.org/data_center/gene_product?id=163340) | 32,491,968 to 32,495,438 | <https://www.maizegdb.org/gene_center/gene?id=GRMZM2G154397> |
|  |  | [col11 - C2C2-CO-like-transcription factor 11](https://www.maizegdb.org/gene_center/gene/col11) | GRMZM2G095598 | 0.17 | CO , CONSTANS-LIKE, constans-like ncsu6, HD1 like, ncsu6, PHM1738, PZA01873, PZB00078, si486056d12, si486056d12, zinc finger protein CONSTANS-LIKE 3-like, ZmBBX5 (per Shalmani, A), ZmCOL04 (per Song, NN) | [B-box; CCT domain protein](https://www.maizegdb.org/data_center/gene_product?id=9040434) | 32,587,885 to 32,589,391 | <https://www.maizegdb.org/gene_center/gene?id=GRMZM2G095598> |
|  |  | [cesa13 - cellulose synthase13](https://www.maizegdb.org/gene_center/gene/cesa13) | GRMZM2G037413 | 3.4 | Cellulose synthase A catalytic subunit 4 [UDP-forming]-like, ZmCESA4 | Cellulose synthase A catalytic subunit 4 [UDP-forming]-like | 29,352,837 to 29,358,720 | <https://www.maizegdb.org/gene_center/gene?id=GRMZM2G037413> |
|  |  | [grx1 - glutaredoxin homolog1](https://www.maizegdb.org/gene_center/gene/grx1) | GRMZM2G150295 | 2.81 | csu40, csu40(glr), csu40(grx), csuh40, umc331 | [glutaredoxin](https://www.maizegdb.org/data_center/gene_product?id=69611) | 29,943,554 to 29,946,951 | <https://www.maizegdb.org/gene_center/gene?id=GRMZM2G150295> |
|  |  | GRMZM2G082191 | GRMZM2G082191 | 2.17 | leucine-rich repeat receptor-like protein kinase, LRR_RI | None | 30,586,576 to 30,591,920 | <https://www.maizegdb.org/gene_center/gene?id=GRMZM2G082191> |
|  |  | [mgt3 - magnesium transporter3](https://www.maizegdb.org/gene_center/gene/mgt3) | GRMZM2G064467 | 1.16 | None | [CorA/MRS2/MGT-type magnesium transporter](https://www.maizegdb.org/data_center/gene_product?id=9034760) | 31,594,459 to 31,599,389 | <https://www.maizegdb.org/gene_center/gene?id=GRMZM2G064467> |
|  |  | [dbb1 - double B-box zinc finger protein1](https://www.maizegdb.org/gene_center/gene/dbb1) | GRMZM2G018876 | 0.93 | AY107218, BBX24 , PCO140184, ZmOrphan198 | [double B-box](https://www.maizegdb.org/data_center/gene_product?id=9036949) | 33,680,677 to 33,725,100 | <https://www.maizegdb.org/gene_center/gene?id=GRMZM2G018876> |
|  |  | [prpo2 - protoporphyrinogen IX oxidase2](https://www.maizegdb.org/gene_center/gene/prpo2) | GRMZM2G364901 | 1.93 | CL1174_1b | [protoporphyrinogen IX oxidase mitochondrial (putative)](https://www.maizegdb.org/data_center/gene_product?id=9030761) | 34,688,186 to 34,701,280 | <https://www.maizegdb.org/gene_center/gene?id=GRMZM2G364901> |
|  |  | [gpdh1 - glucose-6-phosphate dehydrogenase1](https://www.maizegdb.org/gene_center/gene/gpdh1) | GRMZM2G130230 | 3.7 | [csu350(gfu), csu350(gpdh), glucose-6-phosphate 1-dehydrogenase, cytoplasmic isoform, PCO098806](https://www.maizegdb.org/person/1226390) | [glucose-6-phosphate dehydrogenase](https://www.maizegdb.org/data_center/gene_product?id=60682) | 36,456,199 to 36,464,613 | <https://www.maizegdb.org/gene_center/gene?id=GRMZM2G130230> |
|  |  |  |  |  |  |  |  |  |
| **8** | **Chr3_230441690** | [c3h22 - C3H-transcription factor 322](https://www.maizegdb.org/gene_center/gene/c3h22) | GRMZM2G422205 | 4.85 | None | None | 225,587,126 to 225,603,442 | <https://www.maizegdb.org/gene_center/gene?id=GRMZM2G422205> |
|  |  |  |  |  |  |  |  |  |
| **9** | **Chr3_230441714** | [see2b - senescence enhanced2b](https://www.maizegdb.org/gene_center/gene/see2b) | GRMZM2G093032 | 4.62 | PCO131778a, legumain-like protease | None | 225,822,959 to 225,828,669 | <https://www.maizegdb.org/gene_center/gene?id=GRMZM2G093032> |
|  |  | [npf7 - nitrate transporter/peptide transporter family7](https://www.maizegdb.org/gene_center/gene/npf7) | GRMZM2G064091 | 3.36 | NRT1/ PTR FAMILY 6.2, ZmNPF6.2 | [nitrate transporter](https://www.maizegdb.org/data_center/gene_product?id=716017) | 227,085,019 to 227,087,548 | <https://www.maizegdb.org/gene_center/gene?id=GRMZM2G064091> |
|  |  | [IDP798](https://www.maizegdb.org/gene_center/gene/IDP798) | GRMZM2G036050 | 2.82 | CL317_1, CL317_1(279), Cleavage and polyadenylation specificity factor subunit 2 | None | 227,623,191 to 227,632,306 | <https://www.maizegdb.org/gene_center/gene?id=GRMZM2G036050> |
|  |  | [cax3 - calcium exchanger3](https://www.maizegdb.org/gene_center/gene/cax3) | GRMZM2G011592 | 3.25 | AtCAX homolog3, cax3(279), gpm560 (per NCBI), pco087284, rs129589401 (per dbSNP), Vacuolar cation/proton exchanger 3 (per NCBI) | None | 227,189,904 to 227,194,656 | <https://www.maizegdb.org/gene_center/gene?id=GRMZM2G011592> |
|  |  | [osca2 - hyperosmolality-gated calcium-permeable channels2](https://www.maizegdb.org/gene_center/gene/osca2) | GRMZM2G021194 | 2.68 | calcium permeable stress-gated cation channel 1-like , ZmOSCA1.1b | [hyperosmolality-gated calcium-permeable channel protein](https://www.maizegdb.org/data_center/gene_product?id=9042813) | 227,763,311 to 227,774,413 | <https://www.maizegdb.org/gene_center/gene?id=GRMZM2G021194> |
|  |  | [kcs19 - 3-ketoacyl-CoA synthase19](https://www.maizegdb.org/gene_center/gene/kcs19) | GRMZM2G062718 | 2.07 | None | [3-ketoacyl-CoA synthase](https://www.maizegdb.org/data_center/gene_product?id=9039900) | 228,373,735 to 228,375,510 | <https://www.maizegdb.org/gene_center/gene?id=GRMZM2G062718> |
|  |  | [pme31 - pectin methylesterase31](https://www.maizegdb.org/gene_center/gene/pme31) | GRMZM2G004927 | 1.72 | [cl55825_1(280), cl55825_1b, putative pectinesterase 67](https://www.maizegdb.org/person/136031) | [pectinesterase](https://www.maizegdb.org/data_center/gene_product?id=219506) | 228,719,170 to 228,720,718 | <https://www.maizegdb.org/gene_center/gene?id=GRMZM2G004927> |
|  |  | [phot1 - blue-light receptor phototropin 1](https://www.maizegdb.org/gene_center/gene/phot1) | GRMZM2G001457 | 1.31 | CL1834_1, nonphototropic hypocotyl 1 (nph1), NPH, nph1, umc1062, ZmPHOT1 | [serine/threonine protein kinase](https://www.maizegdb.org/data_center/gene_product?id=60686) | 229,132,796 to 229,153,796 | <https://www.maizegdb.org/gene_center/gene?id=GRMZM2G001457> |
|  |  | [asn2 - asparagine synthetase2](https://www.maizegdb.org/gene_center/gene/asn2) | GRMZM2G093175 | 1.02 | AsnS2 | None | 229,419,467 to 229,423,836 | <https://www.maizegdb.org/gene_center/gene?id=GRMZM2G093175> |
|  |  | [rpl10 - ribosomal protein L10 homolog](https://www.maizegdb.org/gene_center/gene/rpl10) | GRMZM2G467086 | 0.9 | [CL12280_1(280), CL12280_1b, uaz198, uaz198a(rpL10), uaz198(L10e)](https://www.maizegdb.org/person/12912) | [ribosomal protein L10e](https://www.maizegdb.org/data_center/gene_product?id=65150) | 229,540,157 to 229,542,366 | <https://www.maizegdb.org/gene_center/gene?id=GRMZM2G467086> |
|  |  | [dmc1 - disrupted meiotic cDNA homolog1](https://www.maizegdb.org/gene_center/gene/dmc1) | GRMZM2G109618 | 0.77 | meiotic recombination protein DMC1 | None | 229,673,847 to 229,678,762 | <https://www.maizegdb.org/gene_center/gene?id=GRMZM2G109618> |
|  |  | [TIDP9234](https://www.maizegdb.org/gene_center/gene/TIDP9234) | GRMZM2G181081 | 0.54 | None | None | 229,904,997 to 229,916,936 | <https://www.maizegdb.org/gene_center/gene?id=GRMZM2G181081> |
|  |  | [gfa1 - glucosamine fructose-6-phosphate aminotransferase1](https://www.maizegdb.org/gene_center/gene/gfa1) | GRMZM2G005849 | 0.44 | [uaz309, uaz309(gfu)](https://www.maizegdb.org/person/12912) | [glucosamine fructose-6-phosphate aminotransferase](https://www.maizegdb.org/data_center/gene_product?id=86832) | 230,000,674 to 230,006,309 | <https://www.maizegdb.org/gene_center/gene?id=GRMZM2G005849> |
|  |  | [gras15 - GRAS-transcription factor 15](https://www.maizegdb.org/gene_center/gene/gras15) | GRMZM5G874545 | 1.61 | None | None | 232,049,326 to 232,071,141 | <https://www.maizegdb.org/gene_center/gene?id=GRMZM5G874545> |
|  |  |  |  |  |  |  |  |  |
| **10** | **Chr6_84376158** | [znfn1 - zinc finger protein1](https://www.maizegdb.org/gene_center/gene/znfn1) | GRMZM2G086277 | 4.88 | [zinc finger, C2H2 type family protein](https://www.maizegdb.org/person/59795) | [zinc-finger transcription factor](https://www.maizegdb.org/data_center/gene_product?id=1271253) | 79,496,641 to 79,498,484 | <https://www.maizegdb.org/gene_center/gene?id=GRMZM2G086277> |
|  |  |  |  |  |  |  |  |  |
| **11** | **Chr6_84376161** | [prc1 - proteasome component1](https://www.maizegdb.org/gene_center/gene/prc1) | GRMZM2G120047 | 3.4 | prc1 proteasome C9 1, uaz237, uaz237b, uaz237b(prc), UAZ237B(Pros) | [proteasome (endopeptidase) component C9](https://www.maizegdb.org/data_center/gene_product?id=65172) | 80,974,657 to 80,975,832 | <https://www.maizegdb.org/gene_center/gene?id=GRMZM2G120047> |
|  |  | [y1 - yellow endosperm1](https://www.maizegdb.org/gene_center/gene/y1) | GRMZM2G300348 | 2.36 | pb1, rs130328408, rs131175743, white1, y1ssr, y4 | [phytoene synthase](https://www.maizegdb.org/data_center/gene_product?id=66643) | 82,017,148 to 82,021,007 | <https://www.maizegdb.org/gene_center/gene?id=GRMZM2G300348> |
|  |  | [mybr98 - MYB-related-transcription factor 98](https://www.maizegdb.org/gene_center/gene/mybr98) | GRMZM2G111906 | 1.88 | [CL42008_-1, cl42008_-1b, tacs1, terminal acidic SANT 1](https://www.maizegdb.org/person/59795) | None | 82,497,057 to 82,500,132 | <https://www.maizegdb.org/gene_center/gene?id=GRMZM2G111906> |
|  |  | [laz8 - lazarus ortholog8](https://www.maizegdb.org/gene_center/gene/laz8) | GRMZM2G373717 | 1.76 | Protein LAZ1, ZmLAZ1-8 | [lazarus](https://www.maizegdb.org/data_center/gene_product?id=9041487) | 82,615,236 to 82,618,088 | <https://www.maizegdb.org/gene_center/gene?id=GRMZM2G373717> |
|  |  | [cyc3 - cyclin3](https://www.maizegdb.org/gene_center/gene/cyc3) | GRMZM2G073671 | 1.76 | [CycIIIZm, umc1186, Zeama;CycB2;1](https://www.maizegdb.org/person/16812) | [cyclin](https://www.maizegdb.org/data_center/gene_product?id=86017) | 82,620,132 to 82,623,958 | <https://www.maizegdb.org/gene_center/gene?id=GRMZM2G073671> |
|  |  | [uaz265b(sbe)](https://www.maizegdb.org/gene_center/gene/uaz265b(sbe)) | GRMZM2G169073 | 1.67 | [1,4-alpha-glucan-branching enzyme, chloroplastic/amyloplastic-like, uaz265b](https://www.maizegdb.org/person/59795) | [starch branching enzyme I](https://www.maizegdb.org/data_center/gene_product?id=97422) | 82,706,565 to 82,725,002 | <https://www.maizegdb.org/gene_center/gene?id=GRMZM2G169073> |
|  |  | [nactf42 - NAC-transcription factor 42](https://www.maizegdb.org/gene_center/gene/nactf42) | GRMZM2G074358 | 1.61 | [nac42, putative NAC domain transcription factor superfamily protein](https://www.maizegdb.org/person/9021409) | none | 82,764,321 to 82,765,776 | <https://www.maizegdb.org/gene_center/gene?id=GRMZM2G074358> |
|  |  | [chn2 - chitinase2](https://www.maizegdb.org/gene_center/gene/chn2) | GRMZM2G145461 | 1.51 | Chit2, chitinase candidateL00973, chn*-L00973, pCh2, uiu5(chn) | [chitinase](https://www.maizegdb.org/data_center/gene_product?id=25130) | 82,862,563 to 82,863,789 | <https://www.maizegdb.org/gene_center/gene?id=GRMZM2G145461> |
|  |  | [bzip15 - bZIP-transcription factor 15](https://www.maizegdb.org/gene_center/gene/bzip15) | GRMZM2G402862 | 0.84 | None | None | 83,537,442 to 83,539,330 | <https://www.maizegdb.org/gene_center/gene?id=GRMZM2G402862> |
|  |  | [bzip106 - bZIP-transcription factor 106](https://www.maizegdb.org/gene_center/gene/bzip106) | GRMZM2G055413 | 0.67 | Basic leucine zipper 19, cl32977_1(73), cl32977_1b | None | 83,703,055 to 83,706,179 | <https://www.maizegdb.org/gene_center/gene?id=GRMZM2G055413> |
|  |  | [shpl2 - shepherd-like2](https://www.maizegdb.org/gene_center/gene/shpl2) | GRMZM2G141931 | 0.6 | endoplasmin | None | 83,777,893 to 83,783,487 | <https://www.maizegdb.org/gene_center/gene?id=GRMZM2G141931> |
|  |  | [cdpk13 - calcium dependent protein kinase13](https://www.maizegdb.org/gene_center/gene/cdpk13) | GRMZM2G173928 | 0.0007 | cdpk*-D84508, ZmCRK3 | [calcium dependent protein kinase](https://www.maizegdb.org/data_center/gene_product?id=56895) | 84,376,922 to 84,383,629 | <https://www.maizegdb.org/gene_center/gene?id=GRMZM2G173928> |
|  |  | [bb9 - double B-box zinc finger protein9](https://www.maizegdb.org/gene_center/gene/dbb9) | GRMZM2G116475 | 0.15 | B-box zinc finger protein 20, salt tolerance protein , ZmBBX23, ZmOrphan149 | [B-box, double B-box](https://www.maizegdb.org/data_center/gene_product?id=9040434) | 84,526,547 to 84,527,970 | <https://www.maizegdb.org/gene_center/gene?id=GRMZM2G116475> |
|  |  | [si1 - silky1](https://www.maizegdb.org/gene_center/gene/si1) | GRMZM2G139073 | 0.41 | at1 (per Patterson, EB), ms-si (per Patterson, EB), PZD00072, si1-at (per Patterson, EB), ts8 (per Stinard, PS), ZmMADS11 ( | None | 84,785,892 to 84,790,566 | <https://www.maizegdb.org/gene_center/gene?id=GRMZM2G139073> |
|  |  | [mybr26 - MYB-related-transcription factor 26](https://www.maizegdb.org/gene_center/gene/mybr26) | GRMZM2G112764 | 0.78 | myb-like transcription factor family protein, si606014a05 | None | 85,157,822 to 85,161,356 | <https://www.maizegdb.org/gene_center/gene?id=GRMZM2G112764> |
|  |  | [srs5 - SHI/STY (SRS)-transcription factor 5](https://www.maizegdb.org/gene_center/gene/srs5) | GRMZM2G108798 | 0.83 | lateral root primordia like7, LRL7, SHI RELATED SEQUENCE 1 | [SHI/STY (SRS)-transcription factor](https://www.maizegdb.org/data_center/gene_product?id=9031348) | 85,205,164 to 85,207,862 | <https://www.maizegdb.org/gene_center/gene?id=GRMZM2G108798> |
|  |  | [arodh3 - arogenate dehydrogenase3](https://www.maizegdb.org/gene_center/gene/arodh3) | GRMZM2G365961 | 1.42 | AroDH-3, pzb01009, rs130332620, rs131175746, rs55623166, ss196416369, ss196416371 | [arogenate dehydrogenase](https://www.maizegdb.org/data_center/gene_product?id=9039320) | 85,796,094 to 85,797,825 | <https://www.maizegdb.org/gene_center/gene?id=GRMZM2G365961> |
|  |  | [pco122535](https://www.maizegdb.org/gene_center/gene/pco122535) | GRMZM2G134759 | 1.51 | pco122535(470), zinc finger (C2H2 type) family protein | None | 85,886,996 to 85,888,733 | <https://www.maizegdb.org/gene_center/gene?id=GRMZM2G134759> |
|  |  | [mpk15 - MAP kinase15](https://www.maizegdb.org/gene_center/gene/mpk15) | GRMZM2G306028 | 1.74 | [gnp_QCH4c06, gpm877, putative mitogen-activated protein kinase 17-3](https://www.maizegdb.org/person/136031) | [MAP kinase (mitogen-activated protein kinase)](https://www.maizegdb.org/data_center/gene_product?id=223021) | 86,113,653 to 86,118,400 | <https://www.maizegdb.org/gene_center/gene?id=GRMZM2G306028> |
|  |  | [eif4a - eukaryotic initiation factor4a](https://www.maizegdb.org/gene_center/gene/eif4a) | GRMZM2G027995 | 2.74 | [gpm272b, IDP1635, ranslational initiation factor eIF-4A, TIDP3537, tif4A, tif-4A3, ucr1a, ucr1a(eif)](https://www.maizegdb.org/person/59795) | [eucaryotic initiation factor 4A](https://www.maizegdb.org/data_center/gene_product?id=112980) | 87,119,198 to 87,123,018 | <https://www.maizegdb.org/gene_center/gene?id=GRMZM2G027995> |
|  |  | mir2(4.5mb) | GRMZM2G150276 | 4.5 | CL872_-2, maize insect resistance1, mir1, mir1(thp) |  | 88909372 to 88911308 | https://www.maizegdb.org/gene_center/gene/mir1 |
|  |  | mir2(4.5mb) | GRMZM2G150256 | 4.5 | CL872_-2, gnp_AW438150, gpm228, maize insect resistance2, mir2, mir2(thp), thp*-Mp708, umc1178 |  | 88903392 to 88908316 | https://www.maizegdb.org/gene_center/gene/mir2 |
|  |  |  |  |  |  |  |  |  |
| **12** | **Chr7_142575520** | [myb152 - MYB-transcription factor 152](https://www.maizegdb.org/gene_center/gene/myb152) | GRMZM2G104551 | 4.37 | cl51309_1, cl51309_1, similar to Arabidopsis myb domain protein 85 (per NCBI), umc1408, ZmMYB111 | None | 143,203,389 to 143,214,173 | <https://www.maizegdb.org/gene_center/gene?id=GRMZM2G104551> |
|  |  | [grftf3 - GRF-transcription factor 3](https://www.maizegdb.org/gene_center/gene/grftf3) | GRMZM2G096709 | 2.59 | cl5295_1, GRF10, GRF10b, GRF10c, grf3 | None | 144,980,649 to 144,983,328 | <https://www.maizegdb.org/gene_center/gene?id=GRMZM2G096709> |
|  |  | [idd7 - indeterminate1 domain7](https://www.maizegdb.org/gene_center/gene/idd7) | GRMZM2G042666 | 1.34 | [AI987507, csh14, ID1 domain7, ID7, iddveg7, si614054G01, veg7](https://www.maizegdb.org/person/1233895) | [indeterminate domain protein](https://www.maizegdb.org/data_center/gene_product?id=952733) | 146,240,249 to 146,245,005 | <https://www.maizegdb.org/gene_center/gene?id=GRMZM2G042666> |
|  |  | [ugp2 - UDP-glucose pyrophosphorylase2](https://www.maizegdb.org/gene_center/gene/ugp2) | GRMZM2G032003 | 1.08 | csu815 | [UDP-glucose pyrophosphorylase](https://www.maizegdb.org/data_center/gene_product?id=66920) | 146,491,263 to 146,498,155 | <https://www.maizegdb.org/gene_center/gene?id=GRMZM2G032003> |
|  |  | [nactf18 - NAC-transcription factor 18](https://www.maizegdb.org/gene_center/gene/nactf18) | GRMZM5G885329 | 0.97 | nac18, NAC domain-containing protein 45 | None | 146,601,629 to 146,605,833 | <https://www.maizegdb.org/gene_center/gene?id=GRMZM5G885329> |
|  |  | [gst16 - glutathione transferase16](https://www.maizegdb.org/gene_center/gene/gst16) | GRMZM5G895383 | 0.25 | CL1110_1, CL1110_1(571) | [glutathione S-transferase subunit 16](https://www.maizegdb.org/data_center/gene_product?id=452075) | 147,829,607 to 147,833,191 | <https://www.maizegdb.org/gene_center/gene?id=GRMZM5G895383> |
|  |  | [camta2 - CAMTA-transcription factor 2](https://www.maizegdb.org/gene_center/gene/camta2) | GRMZM2G032336 | 2.03 | Calmodulin-binding transcription activator 5 | None | 149,602,687 to 149,621,668 | <https://www.maizegdb.org/gene_center/gene?id=GRMZM2G032336> |
|  |  | [myb14 - MYB-transcription factor 14](https://www.maizegdb.org/gene_center/gene/myb14) | GRMZM2G172327 | 2.51 | [R2R3MYB-domain protein, transcription factor MYB21](https://www.maizegdb.org/person/59795) | [myb protein](https://www.maizegdb.org/data_center/gene_product?id=66921) | 150,087,003 to 150,088,438 | <https://www.maizegdb.org/gene_center/gene?id=GRMZM2G172327> |
|  |  | [bub1 - budding inhibited by benzimidazoles homolog1](https://www.maizegdb.org/gene_center/gene/bub1) | GRMZM2G105750 |  | [Mitotic checkpoint serine/threonine-protein kinase, rs130662382 , rs130662533 , rs132415122](https://www.maizegdb.org/person/59795) | [serine/threonine protein kinase](https://www.maizegdb.org/data_center/gene_product?id=60686) | 151,948,871 to 151,964,466 | <https://www.maizegdb.org/gene_center/gene?id=GRMZM2G105750> |
|  |  |  |  |  |  |  |  |  |
| **13** | **Chr8_3163970** | [sbp9 - SBP-transcription factor 9](https://www.maizegdb.org/gene_center/gene/sbp9) | GRMZM2G081127 | 3 | None | squamosa binding protein | 167,824 to 174,330 | <https://www.maizegdb.org/gene_center/gene?id=GRMZM2G081127> |
|  |  | [ms23 - male sterile23](https://www.maizegdb.org/gene_center/gene/ms23) | GRMZM2G021276 | 3.07 | bhlh16 (per Grassius), bHLH-transcription factor 16 (per Grassius), ms35 (per Trimnell, MR), ms*-6011 (per Trimnell, MR), ms*-6018 (per Trimnell, MR), ms*-6027 (per Trimnell, MR), ms*-6031 (per Trimnell, MR), ms*-6059 (per Trimnell, MR), ms*-Bear7, restorer of fertility4 (per Jaqueth, JS), rf4 (per Jaqueth, JS), TIDP2747 (per NCBI) | [bHLH transcription factor](https://www.maizegdb.org/data_center/gene_product?id=9035593) | 95,823 to 98,367 | <https://www.maizegdb.org/gene_center/gene?id=GRMZM2G021276> |
|  |  | [wrky18 - WRKY-transcription factor 18](https://www.maizegdb.org/gene_center/gene/wrky18) | GRMZM2G092694 | 2.51 | WRKY transcription factor 31 | None | 653,032 to 655,605 | <https://www.maizegdb.org/gene_center/gene?id=GRMZM2G092694> |
|  |  | [prpo1 - protoporphyrinogen IX oxidase1](https://www.maizegdb.org/gene_center/gene/prpo1) | GRMZM2G039396 | 2.29 | PCO076390, PCO076390(648), PPO, Zm-ppox | [protoporphyrinogen IX oxidase plastidic](https://www.maizegdb.org/data_center/gene_product?id=315409) | 876,212 to 882,478 | <https://www.maizegdb.org/gene_center/gene?id=GRMZM2G039396> |
|  |  | [mybr2 - MYB-related-transcription factor 2](https://www.maizegdb.org/gene_center/gene/mybr2) | GRMZM2G400489 | 2.51 | myb-related transcription activator | None | 653,032 to 655,605 | <https://www.maizegdb.org/gene_center/gene?id=GRMZM2G092694> |
|  |  | [imd4 - isopropylmalate dehydrogenase4](https://www.maizegdb.org/gene_center/gene/imd4) | GRMZM2G120857 | 0.85 | 3-isopropylmalate dehydrogenase, isocitrate dehydrogenase, IDH, isocitrate dehydrogenase [NAD] catalytic subunit 5 mitochondrial | [isocitrate dehydrogenase; isopropylmalate dehydrogenase](https://www.maizegdb.org/data_center/gene_product?id=13839) | 2,312,133 to 2,319,735 | <https://www.maizegdb.org/gene_center/gene?id=GRMZM2G120857> |
|  |  | [ago4a - argonaute4a](https://www.maizegdb.org/gene_center/gene/ago4a) | GRMZM2G589579 | 0.65 | None | [argonaute](https://www.maizegdb.org/data_center/gene_product?id=978871) | 2,511,663 to 2,519,008 | <https://www.maizegdb.org/gene_center/gene?id=GRMZM2G589579> |
|  |  | [yuc6 - Yucca6](https://www.maizegdb.org/gene_center/gene/yuc6) | GRMZM2G019515 | 0.26 | disulfide oxidoreductase/ monooxygenase/ oxidoreductase | [flavin monoxygenase](https://www.maizegdb.org/data_center/gene_product?id=1232879) | 2,901,119 to 2,904,121 | <https://www.maizegdb.org/gene_center/gene?id=GRMZM2G019515> |
|  |  | [psei4 - cystatin4](https://www.maizegdb.org/gene_center/gene/psei4) | GRMZM2G013461 | 0.18 | [cc4, CC4, cystatin3, multidomain cystatin](https://www.maizegdb.org/person/641780) | [cystatin, cysteine proteinase inhibitor](https://www.maizegdb.org/data_center/gene_product?id=975746) | 3,347,680 to 3,350,552 | <https://www.maizegdb.org/gene_center/gene?id=GRMZM2G013461> |
|  |  | mmp148 | GRMZM2G175071 | 0.26 | hydroxymethylglutaryl-CoA lyase | None | 3,426,738 to 3,431,544 | <https://www.maizegdb.org/gene_center/gene?id=GRMZM2G175071> |
|  |  | [emp7 - empty pericarp7](https://www.maizegdb.org/gene_center/gene/emp7) | GRMZM2G041231 | 1.17 | pentatricopeptide repeat protein, ZmPPR421 | [PPR EMP7 pentatricopeptide repeat protein; PPR pentatricopeptide repeat protein](https://www.maizegdb.org/data_center/gene_product?id=9033983) | 4,334,666 to 4,336,859 | <https://www.maizegdb.org/gene_center/gene?id=GRMZM2G041231> |
|  |  | [bnl13.05a](https://www.maizegdb.org/gene_center/gene/bnl13.05a) | GRMZM2G041308 | 1.18 | peroxidase 72 | None | 4,348,465 to 4,350,696 | <https://www.maizegdb.org/gene_center/gene?id=GRMZM2G041308> |
|  |  | [nactf97 - NAC-transcription factor 97](https://www.maizegdb.org/gene_center/gene/nactf97) | GRMZM2G167492 | 1.38 | nac97, TIP putative NAC domain transcription factor superfamily protein, ZmNTL5 | None | 4,541,745 to 4,558,891 | <https://www.maizegdb.org/gene_center/gene?id=GRMZM2G167492> |
|  |  | [ipp1 - inositol-x-phosphate phosphatase1](https://www.maizegdb.org/gene_center/gene/ipp1) | GRMZM2G029731 | 1.45 | inositol-1-monophosphatase | [inositol-phosphate phosphatase](https://www.maizegdb.org/data_center/gene_product?id=9042609) | 4,615,830 to 4,619,658 | <https://www.maizegdb.org/gene_center/gene?id=GRMZM2G029731> |
|  |  | [c3h28 - C3H-transcription factor 328](https://www.maizegdb.org/gene_center/gene/c3h28) | GRMZM2G036837 | 1.75 | cl10123_1 | None | 4,915,968 to 4,924,817 | <https://www.maizegdb.org/gene_center/gene?id=GRMZM2G036837> |
|  |  | [trps14 - trehalose-6-phosphate synthase14](https://www.maizegdb.org/gene_center/gene/trps14) | GRMZM2G416836 | 2.32 | [protein TPR1, ZmTPS13.1](https://www.maizegdb.org/person/59795) | [trehalose-6-phosphate synthase](https://www.maizegdb.org/data_center/gene_product?id=9022631) | 5,487,619 to 5,499,781 | <https://www.maizegdb.org/gene_center/gene?id=GRMZM2G416836> |
|  |  | [pmei15 - pectin methylesterase inhibitor15](https://www.maizegdb.org/gene_center/gene/pmei15) | GRMZM2G323558 | 2.56 | cl6045_1 | [pectin methylesterase inhibitor](https://www.maizegdb.org/data_center/gene_product?id=9040388) | 5,720,075 to 5,721,677 | <https://www.maizegdb.org/gene_center/gene?id=GRMZM2G323558> |
|  |  | [cys1 - cysteine synthase1](https://www.maizegdb.org/gene_center/gene/cys1) | GRMZM2G082185 | 3.3 | CL5251_1, CL5251_1b, uaz7c02b02(gfu) | [cysteine synthase, plastid](https://www.maizegdb.org/data_center/gene_product?id=104393) | 6,464,565 to 6,468,039 | <https://www.maizegdb.org/gene_center/gene?id=GRMZM2G082185> |
|  |  | [dxr2 - deoxy xylulose reductoisomerase2](https://www.maizegdb.org/gene_center/gene/dxr2) | GRMZM2G036290 | 2.56 | [1-deoxy-D-xylulose 5-phosphate reductoisomerase, chloroplastic, mmp85 (](https://www.maizegdb.org/person/59795) | [1-deoxy-D-xylulose 5-phosphate reductoisomerase](https://www.maizegdb.org/data_center/gene_product?id=9021998) | 5,720,075 to 5,721,677 | <https://www.maizegdb.org/gene_center/gene?id=GRMZM2G036290> |
|  |  | [sin2 - sin homolog2](https://www.maizegdb.org/gene_center/gene/sin2) | GRMZM2G334457 | 4.96 | [paired amphipathic helix protein Sin3-like 4, SIN3 component, histone deacetylase complex, Switch INdependent2, ZmOrphan201, ZmSIN3](https://www.maizegdb.org/person/59795) | [transcription factor](https://www.maizegdb.org/data_center/gene_product?id=62318) | 8,119,203 to 8,129,694 | <https://www.maizegdb.org/gene_center/gene?id=GRMZM2G334457> |
|  |  |  |  |  |  |  |  |  |
| **14** | **Chr8_138888278** | [dof5 - C2C2-Dof-transcription factor 5](https://www.maizegdb.org/gene_center/gene/dof5) | GRMZM5G880268 | 5.05 | bngl2181, bnlg162, bnlg666, ZmDof41 | None | 133,837,595 to 133,839,859 | <https://www.maizegdb.org/gene_center/gene?id=GRMZM5G880268> |
|  |  | [ago1 - argonaute1](https://www.maizegdb.org/gene_center/gene/ago1) | GRMZM2G162525 | 3.98 | [ago113, cl857_3, cl857_3b, gpm630](https://www.maizegdb.org/person/315083) | [argonaute](https://www.maizegdb.org/data_center/gene_product?id=978871) | 134,905,058 to 134,910,463 | <https://www.maizegdb.org/gene_center/gene?id=GRMZM2G162525> |
|  |  | [mmt1 - methionine S-methyltransferase1](https://www.maizegdb.org/gene_center/gene/mmt1) | GRMZM2G098039 | 3.83 | [cl1511_1, mmt1, PZA02033, S-adenosyl-L-methionine:L-methionine S-methyltransferase](https://www.maizegdb.org/person/136031) | [methionine S-methyltransferase](https://www.maizegdb.org/data_center/gene_product?id=923859) | 135,054,057 to 135,061,548 | <https://www.maizegdb.org/gene_center/gene?id=GRMZM2G098039> |
|  |  | [vim102 - variant in methylation102](https://www.maizegdb.org/gene_center/gene/vim102) | GRMZM2G339151 | 3.68 | VIM1-like102 | None | 135,208,445 to 135,212,407 | <https://www.maizegdb.org/gene_center/gene?id=GRMZM2G339151> |
|  |  | [prh19 - protein phosphatase homolog19](https://www.maizegdb.org/gene_center/gene/prh19) | GRMZM2G001243 | 2.87 | probable protein phosphatase 2C 50, ZmOrphan285, ZmPP2C10 | [serine/threonine specific protein phosphatase](https://www.maizegdb.org/data_center/gene_product?id=30136) | 136,017,188 to 136,018,955 | <https://www.maizegdb.org/gene_center/gene?id=GRMZM2G001243> |
|  |  | [wrky68 - WRKY-transcription factor 68](https://www.maizegdb.org/gene_center/gene/wrky68) | GRMZM2G137802 | 2.85 | WRKY7 | None | 136,039,539 to 136,041,895 | <https://www.maizegdb.org/gene_center/gene?id=GRMZM2G137802> |
|  |  | [hdt102 - histone deacetylase102](https://www.maizegdb.org/gene_center/gene/hdt102) | GRMZM2G100146 | 2.76 | gnp_QCI25g10, gpm669, hd2b, HD2b, hda103, PCO120143 | [histone deacetylase](https://www.maizegdb.org/data_center/gene_product?id=173329) | 136,129,273 to 136,131,879 | <https://www.maizegdb.org/gene_center/gene?id=GRMZM2G100146> |
|  |  | [gst27 - glutathione transferase27](https://www.maizegdb.org/gene_center/gene/gst27) | GRMZM2G077206 | 1.98 | [GST 27, PCO061770d](https://www.maizegdb.org/person/9039141) | [glutathione S-transferase subunit 27](https://www.maizegdb.org/data_center/gene_product?id=452087) | 136,911,622 to 136,912,881 | <https://www.maizegdb.org/gene_center/gene?id=GRMZM2G077206> |
|  |  | [ric1 - ras-related protein RIC1](https://www.maizegdb.org/gene_center/gene/ric1) | GRMZM2G106960 | 1.48 | PCO122462, PCO122462(629), Rab1B1, related to ion channel1, ZmRab1B1, ZmRab1B2, ZmRab1B3 | None | 137,403,695 to 137,407,159 | <https://www.maizegdb.org/gene_center/gene?id=GRMZM2G106960> |
|  |  | [see2a - senescence enhanced2a](https://www.maizegdb.org/gene_center/gene/see2a) | GRMZM2G081626 | 1.14 | None | None | 137,751,805 to 137,759,179 | <https://www.maizegdb.org/gene_center/gene?id=GRMZM2G081626> |
|  |  | [mcf2 - mitochrondrial carrier family protein2](https://www.maizegdb.org/gene_center/gene/mcf2) | GRMZM2G420119 | 0.7 | pco110559, pco110559, rs130883616, ZmCoAc1 | [CoA transporter, mitochondrion](https://www.maizegdb.org/data_center/gene_product?id=9024938) | 139,592,349 to 139,595,685 | <https://www.maizegdb.org/gene_center/gene?id=GRMZM2G420119> |
|  |  | [cpd33 - carbohydrate partitioning defective33](https://www.maizegdb.org/gene_center/gene/cpd33) | GRMZM5G852378 | 1.12 | Protein QUIRKY | [multiple C2 domain and transmembrane region protein (MCTP)](https://www.maizegdb.org/data_center/gene_product?id=9041016) | 140,005,748 to 140,009,278 | <https://www.maizegdb.org/gene_center/gene?id=GRMZM5G852378> |
|  |  | [smh1 - single myb histone1](https://www.maizegdb.org/gene_center/gene/smh1) | GRMZM2G136887 | 1.49 | CL35804_1c, fsu1a(smh), hon107, mybr101, MYB-related-transcription factor 101, pUT3449 MYB-related transcription factor (MYBR101), smh101 | [SMH1 (single myb histone1)](https://www.maizegdb.org/data_center/gene_product?id=894674) | 140,378,612 to 140,386,322 | <https://www.maizegdb.org/gene_center/gene?id=GRMZM2G136887> |
|  |  | [ohp3 - opaque2 heterodimerizing protein3](https://www.maizegdb.org/gene_center/gene/ohp3) | GRMZM2G143469 | 1.8 | [pco066337a OHP2, pco066337b](https://www.maizegdb.org/person/59795) | [BZIP protein, o2 heterodimerizing protein](https://www.maizegdb.org/data_center/gene_product?id=9030920) | 140,688,554 to 140,691,786 | <https://www.maizegdb.org/gene_center/gene?id=GRMZM2G143469> |
|  |  | [aldh23 - aldehyde dehydrogenase23](https://www.maizegdb.org/gene_center/gene/aldh23) | GRMZM2G407949 | 1.8 | aldehyde dehydrogenase family 2 member C4, rf2e | [aldehyde dehydrogenase](https://www.maizegdb.org/data_center/gene_product?id=100027) | 140,688,554 to 140,691,786 | <https://www.maizegdb.org/gene_center/gene?id=GRMZM2G407949> |
|  |  | [sun5 - SUN domain protein5](https://www.maizegdb.org/gene_center/gene/sun5) | AC194341.4_FG003 | 2.5 | Sad1p, UNC-84 (snRNP assembly-defective [SAD]; uncoordinated [UNC] = SUN) | None | 141,389,953 to 141,392,153 | <https://www.maizegdb.org/gene_center/gene?id=AC194341.4_FG003> |
|  |  | [mybr35 - MYB-related-transcription factor 35](https://www.maizegdb.org/gene_center/gene/mybr35) | GRMZM2G065829 | 3.06 | putative MYB DNA-binding domain superfamily protein | None | 141,947,729 to 141,950,834 | <https://www.maizegdb.org/gene_center/gene?id=GRMZM2G065829> |
|  |  | [sweet6b - sugars will eventually be exported transporter6b](https://www.maizegdb.org/gene_center/gene/sweet6b) | GRMZM2G416965 | 3.06 | [seven-transmembrane-domain protein 1](https://www.maizegdb.org/person/59795) | [sweet](https://www.maizegdb.org/data_center/gene_product?id=9034268) | 141,947,729 to 141,950,834 | <https://www.maizegdb.org/gene_center/gene?id=GRMZM2G416965> |
|  |  | [sbe3 - starch branching enzyme3](https://www.maizegdb.org/gene_center/gene/sbe3) | GRMZM2G005298 | 3.25 | [BEIII, sbe2a, starch branching enzyme 2a, starch branching enzyme IIa, starch branching enzyme III, umc1997](https://www.maizegdb.org/person/59795) | [starch branching enzyme IIa](https://www.maizegdb.org/data_center/gene_product?id=415180) | 142,143,209 to 142,147,770 | <https://www.maizegdb.org/gene_center/gene?id=GRMZM2G005298> |
|  |  | [sumov1 - small ubiquitin-related modifier-variant1](https://www.maizegdb.org/gene_center/gene/sumov1) | GRMZM2G073404 | 3.25 | [cl7313_1, sumov1(646)](https://www.maizegdb.org/person/136031) | None | 142,143,209 to 142,147,770 | <https://www.maizegdb.org/gene_center/gene?id=GRMZM2G073404> |
|  |  | [mads42 - MADS-transcription factor 42](https://www.maizegdb.org/gene_center/gene/mads42) | GRMZM2G375707 | 3.59 | Agamous-like MADS-box protein AGL62 | None | 142,476,468 to 142,478,055 | <https://www.maizegdb.org/gene_center/gene?id=GRMZM2G375707> |
|  |  | [met4 - DNA methyl transferase4](https://www.maizegdb.org/gene_center/gene/met4) | GRMZM2G157589 | 3.78 | [si687064e06, si687064e06(646), ZmDNMT2 (per Candaele, J), zmet](https://www.maizegdb.org/person/2773369) | [DNA methyl transferase](https://www.maizegdb.org/data_center/gene_product?id=306166) | 142,667,602 to 142,672,558 | <https://www.maizegdb.org/gene_center/gene?id=GRMZM2G157589> |
|  |  |  |  |  |  |  |  |  |
| **15** | **Chr9_113242669** | [hk5 - histidine kinase5](https://www.maizegdb.org/gene_center/gene/hk5) | GRMZM2G025579 | 4.1 | probable histidine kinase 1, Signal-TF , ZmOrphan79 | [Histidine kinase](https://www.maizegdb.org/data_center/gene_product?id=9034927) | 109,142,262 to 109,144,645 | <https://www.maizegdb.org/gene_center/gene?id=GRMZM2G025579> |
|  |  | [bzip17 - bZIP-transcription factor 17](https://www.maizegdb.org/gene_center/gene/bzip17) | GRMZM2G103647 | 4.1 | light-inducible protein CPRF-2 | None | 109,142,262 to 109,144,645 | <https://www.maizegdb.org/gene_center/gene?id=GRMZM2G103647> |
|  |  | [glk34 - G2-like-transcription factor 34](https://www.maizegdb.org/gene_center/gene/glk34) | GRMZM2G081671 | 3.63 | bngl1209, bnlg1209, myb family transcription factor-related protein, ZmPHR17 | None | 109,611,246 to 109,613,880 | <https://www.maizegdb.org/gene_center/gene?id=GRMZM2G081671> |
|  |  | [ca5p7 - CCAAT-HAP5-transcription factor 57](https://www.maizegdb.org/gene_center/gene/ca5p7) | GRMZM2G124421 | 2.79 | si605027f07, ZmNF-YC13, ZmNF-YC15 | [NF-YC, CCAAT-box binding protein subunit C](https://www.maizegdb.org/data_center/gene_product?id=9035479) | 110,455,650 to 110,460,038 | <https://www.maizegdb.org/gene_center/gene?id=GRMZM2G124421> |
|  |  | [mab20 - math-btb20](https://www.maizegdb.org/gene_center/gene/mab20) | GRMZM2G009724 | 2.67 | [speckle-type POZ protein, ZmTRAF5](https://www.maizegdb.org/person/59795) | [BTB/POZ domain protein](https://www.maizegdb.org/data_center/gene_product?id=9025077) | 110,567,873 to 110,569,708 | <https://www.maizegdb.org/gene_center/gene?id=GRMZM2G009724> |
|  |  | [mybr86 - MYB-related-transcription factor 86](https://www.maizegdb.org/gene_center/gene/mybr86) | GRMZM2G170148 | 2.4 | None | None | 110,838,700 to 110,849,958 | <https://www.maizegdb.org/gene_center/gene?id=GRMZM2G170148> |
|  |  | [glk32 - G2-like-transcription factor 32](https://www.maizegdb.org/gene_center/gene/glk32) | GRMZM2G454449 | 1.99 | probable transcription factor KAN4 | None | 111,251,659 to 111,253,947 | <https://www.maizegdb.org/gene_center/gene?id=GRMZM2G454449> |
|  |  | [tubtf13 - TUB-transcription factor 13](https://www.maizegdb.org/gene_center/gene/tubtf13) | GRMZM2G349376 | 1.22 | tub13,tubby-like protein tubby-like F-box protein 11), ZmTLP10 | [tubby-like protein](https://www.maizegdb.org/data_center/gene_product?id=9034113) | 112,018,660 to 112,022,215 | <https://www.maizegdb.org/gene_center/gene?id=GRMZM2G349376> |
|  |  | [bhlh91 - bHLH-transcription factor 91](https://www.maizegdb.org/gene_center/gene/bhlh91) | GRMZM2G049229 | 1.24 | transcription factor MYC2 | None | 112,004,919 to 112,007,822 | <https://www.maizegdb.org/gene_center/gene?id=GRMZM2G049229> |
|  |  | [platz13 - PLATZ-transcription factor 13)](https://www.maizegdb.org/gene_center/gene/platz13) | GRMZM2G093270 | 0.91 | [pco101290, pco101290(682), PLTZ8](https://www.maizegdb.org/person/136031) | None | 112,331,862 to 112,334,178 | <https://www.maizegdb.org/gene_center/gene?id=GRMZM2G093270> |
|  |  | [pap6 - purple acid phosphatase6](https://www.maizegdb.org/gene_center/gene/pap6) | GRMZM2G007754 | 0.01 | [probable inactive purple acid phosphatase 24, ZmPAP2e](https://www.maizegdb.org/person/59795) | [purple acid phosphatase](https://www.maizegdb.org/data_center/gene_product?id=9040053) | 113,231,980 to 113,237,852 | <https://www.maizegdb.org/gene_center/gene?id=GRMZM2G007754> |
|  |  | [pyk3 - pyruvate kinase3](https://www.maizegdb.org/gene_center/gene/pyk3) | GRMZM2G144730 | 0.23 | pco129562 | None | 113,470,642 to 113,476,065 | <https://www.maizegdb.org/gene_center/gene?id=GRMZM2G144730> |
|  |  | [cbl9 - calcineurin B-like9](https://www.maizegdb.org/gene_center/gene/cbl9) | GRMZM2G015324 | 1.21 | [calcineurin B-like protein 9, CBL9](https://www.maizegdb.org/person/59795) | [calcineurin-B like protein](https://www.maizegdb.org/data_center/gene_product?id=9035834) | 114,457,085 to 114,462,156 | <https://www.maizegdb.org/gene_center/gene?id=GRMZM2G015324> |
|  |  | [thx28 - Trihelix-transcription factor 28](https://www.maizegdb.org/gene_center/gene/thx28) | GRMZM2G428470 | 1.31 | trihelix transcription factor ASIL1 | None | 114,551,278 to 114,553,268 | <https://www.maizegdb.org/gene_center/gene?id=GRMZM2G428470> |
|  |  | [mybst1 - single-repeat Myb protein1](https://www.maizegdb.org/gene_center/gene/mybst1) | GRMZM2G034110 | 2.25 | mybr31, MYB-related-transcription factor 31, single-repeat Myb protein, TIDP3321 | [single-repeat Myb protein](https://www.maizegdb.org/data_center/gene_product?id=952737) | 115,490,345 to 115,493,761 | <https://www.maizegdb.org/gene_center/gene?id=GRMZM2G034110> |
|  |  | [cct2 - CO CO-LIKE TIMING OF CAB1 protein domain2](https://www.maizegdb.org/gene_center/gene/cct2) | GRMZM2G004483 | 2.52 | CONSTANS (CO), CO-LIKE and TIMING OF chlorophyll a/b binding protein2 (CAB2), qDTA9(QTL days to anthesis), transcription factor GHD7, ZmCCT9 ZmOrphan327 (per Grassius) | [CCT domain protein](https://www.maizegdb.org/data_center/gene_product?id=9022750) | 115,763,062 to 115,765,952 | <https://www.maizegdb.org/gene_center/gene?id=GRMZM2G004483> |
|  |  | [expb8 - beta expansin8](https://www.maizegdb.org/gene_center/gene/expb8) | GRMZM2G013002 | 3 | csu658(mam) | [expansins](https://www.maizegdb.org/data_center/gene_product?id=275216) | 116,241,684 to 116,244,580 | <https://www.maizegdb.org/gene_center/gene?id=GRMZM2G013002> |
|  |  | [trpp12 - trehalose-6-phosphate phosphatase12](https://www.maizegdb.org/gene_center/gene/trpp12) | GRMZM2G178546 | 3.91 mb | TPP12, TPPA1 , ZmTPP12 | [trehalose-6-phosphate phosphatase](https://www.maizegdb.org/data_center/gene_product?id=9018294) | 117,155,263 to 117,158,898 | <https://www.maizegdb.org/gene_center/gene?id=GRMZM2G178546> |
|  |  | [murE1 - mureinE1](https://www.maizegdb.org/gene_center/gene/murE1) | GRMZM2G009070 | 4.11 | MURE, mureinE homolog1, UDP-N-acetylmuramoyl-L-alanyl-D-glutamate--2,6-diaminopimelate ligase MurE homolog, chloroplastic | [UDP-N-acetylmuramoyl-L-alanyl-D-glutamate---L-lysine ligase](https://www.maizegdb.org/data_center/gene_product?id=9038902) | 117,349,816 to 117,353,530 | <https://www.maizegdb.org/gene_center/gene?id=GRMZM2G009070> |
|  |  | [ppr100 - pentatricopeptide repeat100](https://www.maizegdb.org/gene_center/gene/ppr100) | GRMZM2G428579 | 4.8 | [pentatricopeptide repeat protein PPR566-6, PPR566-6](https://www.maizegdb.org/person/59795) | [PPR pentatricopeptide repeat protein](https://www.maizegdb.org/data_center/gene_product?id=9030899) | 118,045,453 to 118,047,543 | <https://www.maizegdb.org/gene_center/gene?id=GRMZM2G428579> |
|  |  |  |  |  |  |  |  |  |
| **16** | **Chr10_126494378** | [zyp1 - synaptonemal complex protein ZIPPER1](https://www.maizegdb.org/gene_center/gene/zyp1) | GRMZM2G143590 | 4.78 | [synaptonemal complex protein ZYP1, ZEP1, ZIP1](https://www.maizegdb.org/person/16906) | [ZYP1 protein](https://www.maizegdb.org/data_center/gene_product?id=9021424) | 121,717,704 to 121,729,218 | <https://www.maizegdb.org/gene_center/gene?id=GRMZM2G143590> |
|  |  | [serk1 - somatic embryogenesis receptor-like kinase1](https://www.maizegdb.org/gene_center/gene/serk1) | GRMZM5G870959 | 4.74 | PZA03709, PZA03710, PZA03711, PZA03713, rs128615880, rs128615881, rs131176002, rs131176003, rs131176004, ss196417423, ss196417425, ss196417427, ss196417429, ss196417431, ZmSERK1 | [SERK protein](https://www.maizegdb.org/data_center/gene_product?id=403752) | 121,751,970 to 121,757,411 | <https://www.maizegdb.org/gene_center/gene?id=GRMZM5G870959> |
|  |  | [gpa2 - glyceraldehyde-3-phosphate dehydrogenase2](https://www.maizegdb.org/gene_center/gene/gpa2) | GRMZM2G039723 | 4.11 | [csu1047(gpa), csu140, csu140(gpa), gap2, glyceraldehyde-3-phosphate dehydrogenase A, chloroplastic](https://www.maizegdb.org/person/136031) | [glyceraldehyde-3-phosphate dehydrogenase (NADP+) (phosphorylating)](https://www.maizegdb.org/data_center/gene_product?id=13872) | 122,379,626 to 122,381,446 | <https://www.maizegdb.org/gene_center/gene?id=GRMZM2G039723> |
|  |  | [pme1 - pectin methylesterase1](https://www.maizegdb.org/gene_center/gene/pme1) | GRMZM2G125356 | 4.26 | CL1790_1, CL1790_1(750), ZmPME7 | [pectinesterase](https://www.maizegdb.org/data_center/gene_product?id=219506) | 122,230,413 to 122,232,602 | <https://www.maizegdb.org/gene_center/gene?id=GRMZM2G125356> |
|  |  | [grf2 - general regulatory factor2](https://www.maizegdb.org/gene_center/gene/grf2) | GRMZM2G078641 | 3.39 | GF14(14-3-3), ufg8(grf), ufg8(grf1) | [general regulatory factor, 14-3-3 protein](https://www.maizegdb.org/data_center/gene_product?id=65832) | 123,105,640 to 123,116,261 | <https://www.maizegdb.org/gene_center/gene?id=GRMZM2G078641> |
|  |  | [ans2 - anthranilate synthase component II homolog2](https://www.maizegdb.org/gene_center/gene/ans2) | GRMZM2G171383 | 3.2 | anthranilate synthase component II | None | 123,292,346 to 123,296,946 | <https://www.maizegdb.org/gene_center/gene?id=GRMZM2G171383> |
|  |  | [aldh26 - aldehyde dehydrogenase26](https://www.maizegdb.org/gene_center/gene/aldh26) | GRMZM2G135470 | 3.12 | AMADH2, aminoaldehyde dehydrogenase 2, betaine aldehyde dehydrogenase | [aldehyde dehydrogenase](https://www.maizegdb.org/data_center/gene_product?id=100027) | 123,373,488 to 123,378,767 | <https://www.maizegdb.org/gene_center/gene?id=GRMZM2G135470> |
|  |  | [bzip5 - bZIP-transcription factor 5](https://www.maizegdb.org/gene_center/gene/bzip5) | GRMZM2G358701 | 2.83 | transcriptional activator TAF-1-like | None | 123,668,650 to 123,673,853 | <https://www.maizegdb.org/gene_center/gene?id=GRMZM2G358701> |
|  |  | [hagtf41 - GNAT-transcription factor 41](https://www.maizegdb.org/gene_center/gene/hagtf41) | GRMZM2G057554 | 2.81 | [acetyltransferase, GNAT family protein, hag41](https://www.maizegdb.org/person/59795) | None | 123,686,898 to 123,690,430 | <https://www.maizegdb.org/gene_center/gene?id=GRMZM2G057554> |
|  |  | [ras2 - Ras-related protein2](https://www.maizegdb.org/gene_center/gene/ras2) | GRMZM2G173878 | 2.2 | gnp_QAY1g04a, gpm413a, rab2, rab2a, rab2b, rab2-B, Rab2-B, RAB2B, rab2 GTP binding protein, Ras-related protein Rab-2-B, ras*-U22433, "RAt Sarcoma" protein-like, Zm-Rab2-B, ZmRab2-B | None | 124,299,211 to 124,301,978 | <https://www.maizegdb.org/gene_center/gene?id=GRMZM2G173878> |
|  |  | [myb84 - MYB-transcription factor 84](https://www.maizegdb.org/gene_center/gene/myb84) | GRMZM2G173633 | 1.84 | Myb33, putative MYB DNA-binding domain superfamily protein, rs131176006, ss196417435 |  | 124,659,347 to 124,664,396 | <https://www.maizegdb.org/gene_center/gene?id=GRMZM2G173633> |
|  |  | [wrky59 - WRKY-transcription factor 59](https://www.maizegdb.org/gene_center/gene/wrky59) | GRMZM2G031963 | 1.84 | probable WRKY transcription factor 34 | None | 124,659,347 to 124,664,396 | <https://www.maizegdb.org/gene_center/gene?id=GRMZM2G031963> |
|  |  | [nactf15 - NAC-transcription factor 15](https://www.maizegdb.org/gene_center/gene/nactf15) | GRMZM2G111770 | 1.3 | [nac15, SUPPRESSOR OF GAMMA RESPONSE 1](https://www.maizegdb.org/person/9021409) | None | 125,194,444 to 125,197,616 | <https://www.maizegdb.org/gene_center/gene?id=GRMZM2G111770> |
|  |  | [tgz15a - transglutaminase15a](https://www.maizegdb.org/gene_center/gene/tgz15a) | GRMZM2G025054 | 1.19 | [pco063924, pco063924(750)](https://www.maizegdb.org/person/136031) | [transglutaminase](https://www.maizegdb.org/data_center/gene_product?id=952752) | 125,302,195 to 125,306,046 | <https://www.maizegdb.org/gene_center/gene?id=GRMZM2G025054> |
|  |  | [srt101 - sirtuin 101](https://www.maizegdb.org/gene_center/gene/srt101) | GRMZM2G058573 | 0.59 | cl3393_1, cl3393_1(750), hda113, histone deacetylase SIR2 family, NAD-dependent protein deacetylase SRT1, SIR2-like histone deacetylase | [histone deacetylase](https://www.maizegdb.org/data_center/gene_product?id=173329) | 125,905,918 to 125,928,039 | <https://www.maizegdb.org/gene_center/gene?id=GRMZM2G058573> |
|  |  | [hag103b - histone acetyl transferase GNAT/MYST103b](https://www.maizegdb.org/gene_center/gene/hag103b) | GRMZM2G359735 | 0.36 | Elongator complex protein 3, Gcn5-related N-acetyltransferase (GNAT), General Control Nonderepressible (GCN), GNAT-transcription factor 42, hac107, hagtf42 | [histone acetyltransferase GNAT/MYST](https://www.maizegdb.org/data_center/gene_product?id=173330) | 126,138,815 to 126,144,100 | <https://www.maizegdb.org/gene_center/gene?id=GRMZM2G359735> |
|  |  | [thx25 - Trihelix-transcription factor 25](https://www.maizegdb.org/gene_center/gene/thx25) | GRMZM2G047370 | 0.004 | None | None | 126,489,800 to 126,493,558 | <https://www.maizegdb.org/gene_center/gene?id=GRMZM2G047370> |
|  |  | [nlp9 - NLP-transcription factor 9](https://www.maizegdb.org/gene_center/gene/nlp9) | GRMZM2G105004 | 1.08 | AY110634, CL3121_1, nip9, ZmNLP2.1, ZmNLP9 | [RWP-RK domain containing protein](https://www.maizegdb.org/data_center/gene_product?id=9039224) | 127,570,133 to 127,575,182 | <https://www.maizegdb.org/gene_center/gene?id=GRMZM2G105004> |
|  |  | [sam1 - S-adenosylmethionine decarboxylase1](https://www.maizegdb.org/gene_center/gene/sam1) | GRMZM2G125635 | 3.09 | csu217, csu217(gfu), csu6B, csu6b(sam), csuh6, S-adenosylmethione decarboxylase1, stm*-csu217, umc305 | [S-adenosylmethionine decarboxylase](https://www.maizegdb.org/data_center/gene_product?id=163340) | 129,584,660 to 129,588,101 | <https://www.maizegdb.org/gene_center/gene?id=GRMZM2G125635> |
|  |  | [mybr68 - MYB-related-transcription factor 68](https://www.maizegdb.org/gene_center/gene/mybr68) | GRMZM2G410083 | 3.09 | [transcription factor MYB2, umc1678](https://www.maizegdb.org/person/59795) | None | 129,584,660 to 129,588,101 | <https://www.maizegdb.org/gene_center/gene?id=GRMZM2G410083> |
|  |  | [amt1 - ammonium transporter1](https://www.maizegdb.org/gene_center/gene/amt1) | GRMZM2G175140 | 3.58 | [ZmAMT1;1a, ZmAMT1.1A](https://www.maizegdb.org/person/9023996) | [ammonium transporter protein](https://www.maizegdb.org/data_center/gene_product?id=9024003) | 130,079,195 to 130,081,274 | <https://www.maizegdb.org/gene_center/gene?id=GRMZM2G175140> |
|  |  | [nactf65 - NAC-transcription factor 65](https://www.maizegdb.org/gene_center/gene/nactf65) | GRMZM2G043813 | 4.13 | nac65 | None | 130,620,219 to 130,622,989 | <https://www.maizegdb.org/gene_center/gene?id=GRMZM2G043813> |
|  |  | [myb132 - MYB-transcription factor 132](https://www.maizegdb.org/gene_center/gene/myb132) | AC206901.3_FG005 | 4.23 | None | None | 130,721,588 to 130,723,152 | <https://www.maizegdb.org/gene_center/gene?id=AC206901.3_FG005> |
|  |  | [arftf38 - ARF-transcription factor 38](https://www.maizegdb.org/gene_center/gene/arftf38) | GRMZM2G005284 | 4.23 | [arf38, auxin response factor 10](https://www.maizegdb.org/person/9021409) | None | 130,721,588 to 130,723,152 | <https://www.maizegdb.org/gene_center/gene?id=GRMZM2G005284> |
